# Ovary Abortion Induced by Combined Waterlogging and Shading Stress at the Flowering Stage Involves Amino Acids and Flavonoid Metabolism in Maize

**DOI:** 10.3389/fpls.2021.778717

**Published:** 2021-11-23

**Authors:** Jinlong Zhou, Lei Tian, Shunxi Wang, Hongping Li, Yali Zhao, Moubiao Zhang, Xiuling Wang, Panpan An, Chaohai Li

**Affiliations:** College of Agronomy, Henan Agricultural University, Zhengzhou, China

**Keywords:** combined waterlogging and shading, ovary, silk, abortion, carbon, amino acid, flavonoid

## Abstract

Maize (*Zea mays* L.) crops on the North China Plain are often subject to continuous overcast rain at the flowering stage. This causes waterlogging and shading stresses simultaneously and leads to huge yield losses, but the causes of these yield losses remain largely unknown. To explore the factors contributing to yield loss caused by combined waterlogging and shading stress at the flowering stage, we performed phenotypic, physiological, and quasi-targeted metabolomics analyses of maize plants subjected to waterlogging, shading, and combined waterlogging and shading (WS) treatments. Analyses of phenotypic and physiological indexes showed that, compared with waterlogging or shading alone, WS resulted in lower source strength, more severe inhibition of ovary and silk growth at the ear tip, a reduced number of emerged silks, and a higher rate of ovary abortion. Changes in carbon content and enzyme activity could not explain the ovary abortion in our study. Metabolomic analyses showed that the events occurred in ovaries and silks were closely related to abortion, WS forced the ovary to allocate more resources to the synthesis of amino acids involved in the stress response, inhibited the energy metabolism, glutathione metabolism and methionine salvage pathway, and overaccumulation of H_2_O_2_. In silks, WS led to lower accumulation levels of specific flavonoid metabolites with antioxidant capacity, and to over accumulation of H_2_O_2_. Thus, compared with each single stress, WS more seriously disrupted the normal metabolic process, and resulted more serious oxidative stress in ovaries and silks. Amino acids involved in the stress response in ovaries and specific flavonoid metabolites with antioxidant capacity in silks play important roles during ovary abortion. These results identify novel traits for selection in breeding programs and targets for genome editing to increase maize yield under WS stress.

## Introduction

Maize (*Zea mays* L.) is one of the most important crops in the world, serving as an essential source of feed, food, biomass for energy production, and as an industrial raw material. The period before and after silking is the most sensitive period of maize, and is crucial in determining the final grain number (Boyer, 2010; Li et al., 2018). Maize ovary development is also highly environmentally sensitive. Abiotic stress during the flowering period results in early abortion, and the reasons for early grain abortion differ depending on the type and extent of the abiotic stress (Hütsch et al., 2014; Oury et al., 2016b; Jung et al., 2017; Shen et al., 2018; Wang et al., 2020). Some previous studies found that a decrease in acid invertase activity was associated with limited kernel setting under salt stress (Hütsch et al., 2014), and that changes in CWI activity under drought stress were related to ovary abortion (Boyer, 2004; McLaughlin and Boyer, 2004; Chen et al., 2019). However, other studies found that, under moderate drought stress during the flowering period, ovary abortion was not determined by changes in sugar concentrations or invertase activity (Henry et al., 2015; Turc and Tardieu, 2018). In another study, the sugar and starch concentrations decreased in ovaries under shading stress from 3–4 days after silk emergence, but abortion did not occur (Hiyane et al., 2010). Therefore, even though the grain abortion rate is higher under abiotic stress conditions during the flowering period in maize, the relationship between abortion and carbon status is unclear, and other factors may contribute to this process.

Summer maize on the North China Plain is often subjected to continuous overcast rain during the flowering period, resulting in yield losses of more than 20% (Zhang et al., 2009; Feng et al., 2011; Cui et al., 2012; An et al., 2018). The main consequence of continuous overcast rain is that plants are subjected to combined waterlogging and shading, which seriously affects maize development and reproduction. As a typical C4 plant, maize is very sensitive to shading (Bellasio and Griffiths, 2014). A previous study showed that shading lengthens the ASI, reduces dry matter accumulation, affects silk differentiation and setting percentage, damages mesophyll cell ultrastructure, reduces the photosynthetic rate, and decreases GY (Jing et al., 2009; Cui et al., 2015; Ren et al., 2016a). Shading has also been shown to affect carbon and nitrogen metabolism in maize, leading to the accumulation of amino acids and proteins related to stress/defense/detoxification, and decreased abundance of proteins related to starch and sucrose metabolism, glycolysis, and the TCA cycle (Jing et al., 2009; Ren et al., 2016a; Cotrozzi and Landi, 2018; Gao et al., 2020; Liang et al., 2020; Wang et al., 2020). Maize is very sensitive to waterlogging at the seedling stage but strongly resistant after the flowering stage. Waterlogging damages photosynthetic systems and the antioxidant system, leading to the accumulation of ROS. It also affects nitrogen metabolism, and decreases in nitrogen absorption and transportation negatively affect grain yield (Ren et al., 2016b, 2017, 2021). Physiological and proteomic analyses have shown that waterlogging leads to increased concentrations of ethylene and polyamines in maize leaves, disrupts energy metabolism, and decreases photosynthesis as a result of ROS accumulation (Chen et al., 2014). Waterlogging affects dry matter translocation from leaves and stems to ears in maize, resulting in insufficient grain filling. Genes involved in protein degradation, signal transduction, and carbon metabolism play important roles in the adaptation to waterlogging stress (Kaur et al., 2021). Despite the severe effects of waterlogging and shading on maize, previous studies have mainly focused on single waterlogging or shading stresses. Moreover, ovary growth and the rapid extension of silks in maize at the flowering stage are crucial for kernel setting, but few studies have focused on how these processes are affected by waterlogging, shading, and the combination of these stresses.

Understanding the complexity of the mechanisms responsible for ovary and silk development and how waterlogging and shading stress cause ovary abortion is critically important to improve maize production. Metabolomic studies have unraveled some mechanisms underlying kernel development (Wen et al., 2014; Yang et al., 2018; Bernardi et al., 2019; Gálvez Ranilla, 2020) and the responses of different crop varieties to various abiotic stress (Obata et al., 2015; Sun et al., 2016; Xiong et al., 2019; Wang et al., 2021). The reasons for maize ovary/kernel abortion in response to abiotic stress are very complex. Most previous studies have focused on the relationship between abortion and carbon status, but this relationship is still unclear. Ovary/kernel abortion may be related to changes in other metabolites or metabolic pathways. To date, no previous studies have conducted metabolome analyses of maize subjected to waterlogging, shading, and the combination of these two stresses at the flowering stage.

The aim of this study was to explore the process of ovary/kernel abortion and how it relates to carbon status. To this end, we analyzed the growth and development characteristics, physiological indices, carbon status, and quasi-targeted metabolome of maize under single and combined waterlogging and shading stresses. The metabolic characteristics of maize under single and combined waterlogging and shading stresses were determined to identify metabolites and metabolic pathways closely related to ovary abortion. This is the first metabolome study of the response of maize to these stresses, alone and combined, at the flowering stage, with a specific focus on the metabolic changes associated with ovary abortion. The results of this study have potential applications in molecular breeding and in biotechnological strategies to improve the stress resistance and yield of maize.

## Materials and Methods

### Plant Materials and Treatments

The maize commercial variety Yuyu22 was grown in pots in a 3.5-m high isolation chamber at Henan Agricultural University (Zhengzhou, China). The test pots were cylindrical, 35 cm high, with top and bottom diameters of 32 cm and 27 cm, respectively. The soil samples were collected from the plowed layer (0–20 cm), sieved, and mixed well. Each pot was filled with 15 kg soil. The soil properties were as follows: organic matter 10.2 g/kg, available nitrogen 75.3 mg/kg, available phosphorus 25.5 mg/kg, and available potassium 161.4 mg/kg. Three seeds were sown in each pot, and the seedlings were then thinned to one per pot. The pots were irrigated regularly to maintain optimum soil moisture until the waterlogging treatments were applied. When the tassels had fully emerged from the whorl, healthy uniform plants were selected and randomly allocated to four groups: control (CK), waterlogging stress (W), shading stress (S), and combined waterlogging and shading stress (WS) treatments. In the W and WS treatments, the water level was maintained at 2–3 cm above the soil surface until 3 days after pollination (3DAP). In the S and WS treatments, plants were shaded with black polypropylene fabric with 50% light penetration during the same period. The plants in CK had normal light and irrigation conditions. The time course of the stress treatments is shown in Supplementary Figure 1. To test whether other environmental factors affected the results, microclimate data were recorded every day during the treatment period at 11:00 AM (Supplementary Table 1). In all groups, ears were bagged with paper bags before silk emergence and hand-pollinated with fresh pollen from control plants at the day of full silk emergence.

### Plant Sampling and Measurements

Ears and ear leaves were sampled at 10 AM from plants at three stages: (1) SE, first silk emergence; (2) P, full silk emergence, and (3) 3DAP. Samples were collected from six maize plants in each group (CK, W, S, and WS) at the three stages (i.e., six biological replicates per treatment at each sampling time). Ear leaves were sampled with a hole punch, at the middle position, and the samples were frozen in liquid nitrogen. The ear was then dissected, and the ear length and the fresh weights of the husks, peduncles, ears, and silks were measured immediately. Silks were sampled from positions 30–45 along the ear rows, and the sampled silks were directly frozen in liquid nitrogen. The ovaries were sampled from positions 30–45 along each side of the ear rows. The total weight was divided by 32 to obtain the fresh ovary weight, then the ovaries were frozen in liquid nitrogen. The sampled leaves, ovaries, and silks were stored at −80°C until further analyses. The length and number of emerged silks were determined at 6.00 PM from SE until the day that silks stopped growing. The length of newly emerged silks extending from bract was measured on the first day of silk emergence at 6 PM, then the emerged silks were cut from the apex of the bracts, and the length of the newly emerged silks on the same plants was recorded at the same time the next day (SE1). This procedure was repeated until no more silks extended from the bract. The silk traits were measured on unpollinated plants. Yield data were calculated from the weight of dry seeds at the fully ripe stage.

### Dry Matter Accumulation and Proportion of Biomass Allocated to Ears

Three representative plants were collected at SE, P, and 3DAP, and separated into shoots, roots, and ears. These samples were oven-dried to constant weight at 80°C in a forced draft oven and then weighed. The PBE was calculated as follows: ear dry weight ÷ shoot dry weight.

### Photosynthesis Measurements and SPAD Chlorophyll Value

A portable photosynthesis system (Li-6400XT, LI-COR, Lincoln, NE, United States) was used to measure the net photosynthetic rate (*P*_*n*_), stomatal conductance (*G*_*s*_), transpiration rate (*T*_*r*_), and intercellular CO_2_ concentration (*C*_*i*_). These measurements were conducted on the ear leaf of four different plants between 11.00 AM and 2.00 PM for plants in CK and all treatment groups at SE, P, and 3DAP. The settings were as follows: Ref CO_2_: 400 μmol mol^–1^, photosynthetically active radiation: 1400 μmol m^–2^ s^–1^ for the CK and W groups, and 700 μmol m^–2^ s^–1^ for the S and WS groups. The chlorophyll SPAD value was measured on the ear leaf at SE, P, and 3DAP in six randomly selected plants per treatment using a portable chlorophyll meter.

### Determination of Sugar Contents

Three biological replicates were randomly selected from leaf, ovary, and silk samples. The samples were ground in liquid nitrogen, and then 100 mg ground tissue was used for the determination of sugar contents. Sucrose, glucose, and fructose contents in ethanolic extracts were determined as described by Hendriks et al. (2003). Starch was extracted from the pellets of the ethanol extracts. The pellets were solubilized in 0.1 M NaOH by heating to 95°C for 30 min, and then starch was quantified by measuring the amount of glucose released *via* hydrolysis.

### Measurement of Enzyme Activities and H_2_O_2_ Content

Three biological replicates were randomly selected from leaf, ovary, and silk samples. The samples were ground in liquid nitrogen and then 10 mg ground tissue was used for the determination of enzyme activities. Then extracts were prepared as described by Zinselmeier et al. (1999). The activity of SPS was measured according to the method described by Gibon et al. (2009). The activities of CWI and VI were measured as described by Wang et al. (2010). The activity of SuSy was determined as described by Ruan et al. (2003).

To determine H_2_O_2_ content, 100-mg samples of leaves, ovaries, and silks, were ground in liquid nitrogen, homogenized in an ice bath with 1 mL 0.1% v/v trichloroacetic acid, and then centrifuged at 12,000 × *g* at 4°C for 15 min. A 500 μL aliquot of the supernatant was added to 0.5 mL 10 mM potassium phosphate buffer (pH 7.0) and 1 mL 1 M KI, then H_2_O_2_ was quantified as described by Velikova et al. (2000).

### Quasi-Targeted Metabolomic Analyses

Quasi-targeted metabolomics analyses was performed by Novogene Bioinformatics Technology Co., Ltd. (Beijing, China). Samples of leaves, ovaries, and silks (100 mg) were individually ground with liquid nitrogen. The homogenate was mixed with 500 μL 80% prechilled methanol and 0.1% formic acid by vortexing. The samples were incubated on ice for 5 min and then centrifuged at 15,000 × *g* at 4°C for 10 min. An aliquot of the supernatant was diluted to a final concentration of 53% methanol with LC-MS grade water. The samples were subsequently transferred to a fresh Eppendorf tube and then centrifuged at 15,000 × *g* at 4°C for 20 min. Finally, the supernatant was injected into an LC-MS/MS system for analyses. Detailed methods and procedures including instrument parameters, data acquisition and processing, metabolite identification and quantification, data normalization, and statistical analyses can be found in Supplementary File 1.

### Weighted Gene Co-expression Network Analysis

Relationships between compounds in the leaf, ovary, and silks identified in the quasi-targeted metabolomics analyses and agronomic and physiological characteristics, sugar content, and enzyme activities were analyzed using the R package weighted gene co-expression network analysis (WGCNA). The WGCNA was performed according to Langfelder and Horvath (2008). A β-soft power threshold of 12 was selected to ensure that the network satisfied a scale-free topology (*R*^2^ > 0.9) based on the linear regression model fitting index obtained from the functions “pickSoftThreshold” operation. Co-expression modules were detected using the function ‘blockwiseModules’ with default settings (minModuleSize, 50; mergeCutHeight, 0.25).

### Statistical Analyses

All measurements in this study were conducted with at least three biological replicates. Analysis of variance (ANOVA) and least significant difference (LSD) tests were conducted using SPSS software (Ver. 22.0, IBM Corp., Armonk, NY, United States). Figures were produced with Sigma Plot 14.0. Data presented in bar charts were subjected to one-way ANOVA followed by Duncan’s multiple range test, with statistical significance accepted at *P* < 0.05. Figures were constructed using PowerPoint and Photoshop (Adobe Photoshop CC 2017) software.

## Results

### Combined Waterlogging and Shading Impaired Maize Growth and Reproduction More Strongly Than Did Waterlogging or Shading

In this study, the commercial maize variety Yuyu22 was subjected to W, S, and WS treatments from tassel emergence until 3DAP, and the plants in CK received normal light and irrigation. The data for maize growth at flowering period and yield are shown in Table 1. Ear FW, ear length, shoot DW, and root DW were measured at SE, P, and 3DAP. The ASI extended from 2.4 d in CK to 3.3 d, 3.6 d, and 4.3 d in the W, S, and WS groups, respectively (Table 1). Ear FW, ear length, shoot DW, and root DW were significantly decreased in all stress groups compared with CK, and were lowest in WS. The KN was reduced by 21%, 36%, and 57% in the W, S, and WS groups, respectively, compared with that in CK, but the 100-grain weight was not affected, showing that the yield loss was due to the reduced KN rather than reduced grain weight. This indicated that a large number of ovaries (before fertilization) or kernels (after fertilization) were aborted in the W, S, and WS treatments. Taken together, these results showed that compared with single W or S treatments, the WS treatment had more serious effects on the growth and reproduction of maize, leading to a higher abortion rate of ovaries/kernels.

**TABLE 1:** Phenotypes of maize plants in control (CK), waterlogging + shading (WS), waterlogging (W), and shading (S) treatment groups.

**Treatment**	**Stage**	**ASI**	**Ear FW**	**Ear length**	**Shoot DW**	**Root DW**	**Kernel number (per ear)**	**100-grain weight (g)**
		*d*	*G*	*cm*	*g*	*g*		*g*
CK	SE	2.43 ± 0.5	17.28 ± 3.35	11.75 ± 0.86	143.22 ± 5.33	21.52 ± 0.46	687.67 ± 22.27	25.71 ± 0.95
	P		60.37 ± 2.76	17.3 ± 0.34	166.88 ± 1.29	24.43 ± 0.43		
	3DAP		159.67 ± 18.11	21.15 ± 0.68	185.11 ± 3.08	27.9 ± 0.67		
W	SE	**3.3 ± 0.71**	15.45 ± 1.08	11.12 ± 0.41	125.88 ± 2.48	21.22 ± 0.49	**544.22 ± 31.34**	25.96 ± 0.93
	P		**49.38 ± 4.83**	**15.37 ± 0.46**	**141.64 ± 3.3**	**22.86 ± 0.19**		
	3DAP		**136.46 ± 5.43**	**18.93 ± 0.78**	**161.83 ± 3.17**	**25.61 ± 0.31**		
S	SE	**3.6 ± 1.01**	**11.89 ± 0.93**	**9.92 ± 0.48**	**115.91 ± 3.34**	**20.36 ± 0.33**	**437.89 ± 28.59**	26.06 ± 0.68
	P		**36.35 ± 5.11**	**14.57 ± 0.93**	**123.12 ± 2.06**	**22.53 ± 0.47**		
	3DAP		**70.12 ± 3.36**	**16.52 ± 0.39**	**135.65 ± 1.76**	**25 ± 0.56**		
WS	SE	**4.26 ± 1.08**	**11.19 ± 1.57**	**9.73 ± 0.5**	**106.19 ± 1.18**	**19.77 ± 0.23**	**292.44 ± 15.36**	26.25 ± 1.00
	P		**17.61 ± 4.94**	**11.38 ± 0.87**	**112.65 ± 3.78**	**21.43 ± 0.41**		
	3DAP		**51.76 ± 3.52**	**12.87 ± 1.41**	**123.79 ± 2.76**	**22.48 ± 0.44**		
	*n*	38–44	6	6	3	3	9	9

*For ear FW (fresh weight), ear length, shoot DW (dry weight), and root DW, developmental stage is precise. Kernel number and 100-grain weight were measured at maturity. Values in bold are significantly different from the control (*P* < 0.05), underlined values are significantly different from the single waterlogging and shading treatment (*P* < 0.05). Values are means ± SD.*

*ASI (anthesis silking interval), SE (first silk emergence), P (full silk emergence), 3DAP (3 days after pollination).*

### Compared With Single Stresses, Combined Waterlogging and Shading More Strongly Inhibited Photosynthesis and Root Absorption Ability

**FIGURE 1:**
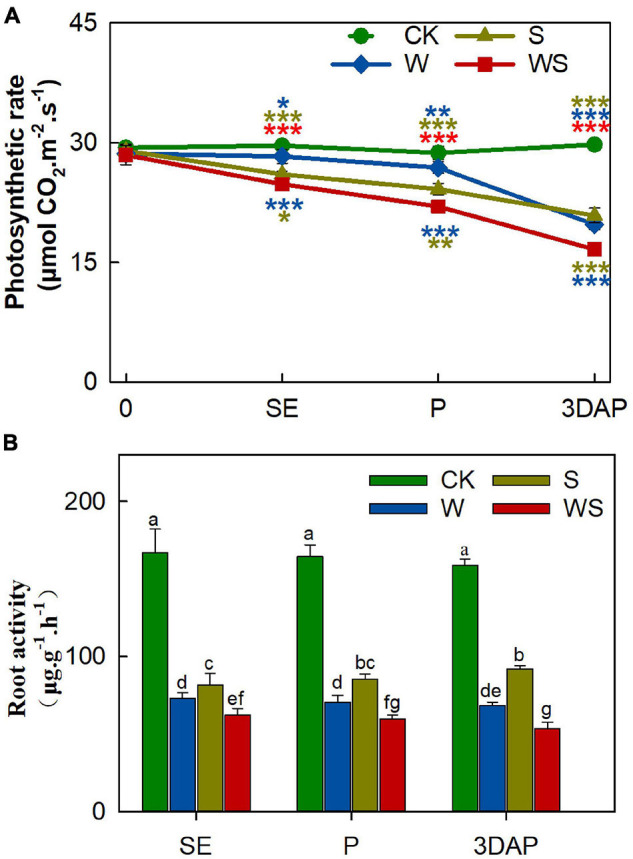
Photosynthetic rate **(A)** and root activity **(B)** of plants in control (CK), waterlogging + shading (WS), waterlogging (W), and shading (S) treatment groups. Photosynthetic rate was taken at start of all treatments (0) and at SE (first silk emergence), P (full silk emergence), and 3DAP (3 days after pollination). Root activity was taken at SE, P, and 3DAP. Data were subjected to one-way ANOVA followed by Duncan’s new multiple range test. Different letters above bars indicate significant differences. Colored asterisks above line chart indicate significant differences between CK and treatment groups, colored asterisks below indicate significant differences between WS and W or S groups. Error bars represent ± SD (*n* ≥ 4). *P < 0.05, **P < 0.01, ***P < 0.001 (ANOVA and *t*-test).

To explore the effects of the W, S, and WS treatments on photosynthesis and root absorption ability, we measured the *P*_*n*_, *T*_*r*_, *C*i, *G*_*s*_, chlorophyll SPAD, and root activity. The *P*_*n*_ value significantly decreased in all treatment groups from SE onward, and the difference in *P*_*n*_ values between the treatment groups and CK gradually became larger over time (Figure 1A). The *P*_*n*_ values were significantly lower in the WS group than in the W and S groups. Root activity was severely inhibited in all treatment groups from SE onward, and was significantly lower in the WS group than in the W or S groups (Figure 1B). The *T*_*r*_ values were lower in the W and WS groups than in the S group (Supplementary Figure 2A). The trends in *T*_*r*_ were similar in the W and WS groups, but the *T*_*r*_ value was lower in the W group than in the WS group at 3DAP. The *C*_*i*_ values were higher in the S group than in CK, and the decline in *C*_*i*_ showed similar trends in the W and WS groups (Supplementary Figure 2B). The changes in *G*_*s*_ were similar to the changes in *T*_*r*_ (Supplementary Figure 2C). The SPAD values were lower in the W and WS groups than in CK, but not significantly different between the S group and CK (Supplementary Figure 2D). The results indicated that photosynthetic characteristics, especially *P*_*n*_, and root activity were significantly affected by these stress treatments. The values for *P*_*n*_ and root activity were lower in all treatment groups than in CK and were lowest in the WS group. Thus, the WS treatment affected the uptake and assimilation abilities of maize source tissues more strongly than did the W or S treatments.

### Ovary and Silk Growth Were More Strongly Inhibited by Combined Waterlogging and Shading Than by Waterlogging or Shading, Resulting in Increased Ovary Abortion

**FIGURE 2:**
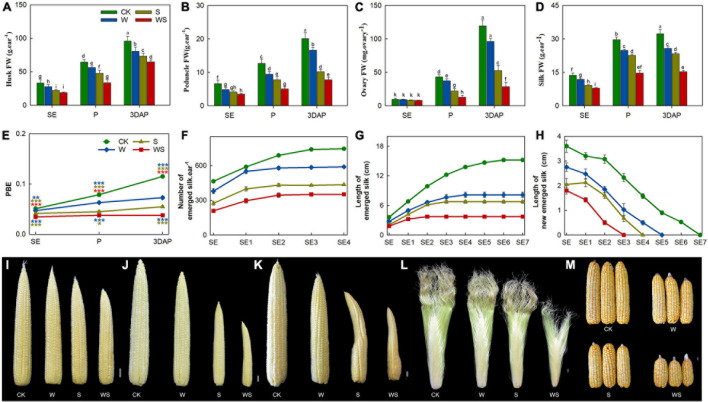
Growth status and phenotype of ear in control (CK), waterlogging + shading (WS), waterlogging (W), and shading (S) treatment groups. Husks fresh weight (FW) **(A)**, peduncle FW **(B)**, ovaries FW (from positions 30–45 along the ear rows) **(C)**, silks FW **(D)**, proportion of biomass allocation to ear (PBE) **(E)**, total number of emerged silks every day **(F)**, length of emerged silk from bract every day **(G)**, length of newly emerged silk from bract per day **(H)**. Phenotypes of ears at SE (first silk emergence) **(I)**, P (full silk emergence) **(J,L)**, 3DAP (3 days after pollination) **(K)** and harvest **(M)**. Data were subjected to one-way ANOVA followed by Duncan’s new multiple range test. Different letters above bars indicate significant differences. Colored asterisks above line chart indicate significant differences between CK and treatment groups, colored asterisks below indicate significant differences between WS and W or S groups. Error bars represent ± SD (*n* ≥ 4). *P < 0.05, **P < 0.01, ***P < 0.001 (ANOVA and *t*-test).

Next, we analyzed various parameters of reproductive organs. The FW of husks, peduncles, and silks were significantly lower in all treatment groups than in CK from SE onward, while the ovary FW was decreased in all treatment groups from P onward (Figures 2A–D). In addition, the FWs of the husk, peduncle, and ovary were significantly lower in the WS group than in the W or S groups from P onward, and the silk FW was significantly lower in the WS group than in the W and S groups from SE onward. The proportion of biomass allocated to the ear (PBE) was significantly lower in all treatment groups than in CK from SE onward, and was decreased by 37%, 53%, and 67% at 3DAP in the W, S, and WS groups, respectively (Figure 2E). The growth conditions and number of emerged silks are crucial factors determining fertilization and KN, so continuous measurements were performed for emerged silks. The total number of emerged silks was significantly lower in the treatment groups than in CK from SE onward. Thus, the final numbers of emerged silks were 21%, 42%, and 53% lower in the W, S, and WS groups, respectively, than in CK (Figure 2F). The length of newly grown emerged silks per day and the overall emerged silk length were significantly lower in the treatment groups than in the control and were lowest in the WS group. The growing period of silks was 7, 5, 4, and 3 days in CK, W, S, and WS groups, respectively (Figures 2G,H). In the treatment groups, the phenotype of the ear was obviously affected, with the most severely affected ears in the WS group (Figures 2I–L). Finally, large-scale ovary abortion concentrated at the ear tip occurred in the treatment groups (Figure 2M). A correlation analysis revealed that the final number of emerged silks was highly consistent with KN (Supplementary Figure 3). Therefore, compared with W or S, WS had more serious effects on ear tissues (including FW, PBE, silk number and elongation). Aborted ovaries were concentrated at the tip ear in all treatment groups, and the final silk number was highly correlated with KN.

### Differences in Sugar Content and Enzyme Activities in Source and Sink Organs Among Waterlogging, Shading, and Combined Waterlogging and Shading Groups

**FIGURE 3:**
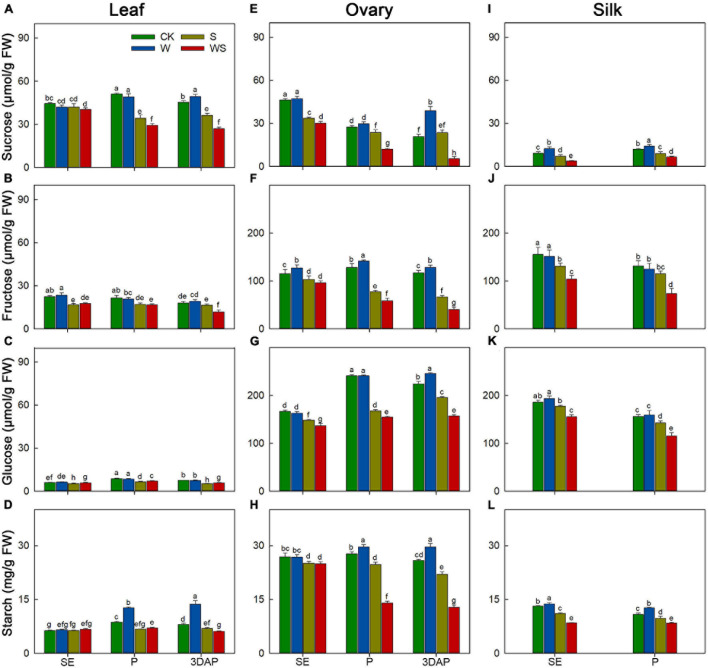
Changes in the contents of sucrose, fructose, glucose, and starch in leaf **(A–D)**, ovary **(E–H)** and silk **(I–L)** at SE (first silk emergence), P (full silk emergence), and 3DAP (3 days after pollination) in control (CK), waterlogging + shading (WS), waterlogging (W), and shading (S) treatment groups. Data were subjected to one-way ANOVA followed by Duncan’s new multiple range test (*n* = 3). Different letters above bars indicate significant differences (*P* < 0.05). Error bars represent ± SD.

Next, we determined the effects of the stress treatments on the contents of carbohydrates (sucrose, fructose, glucose, and starch) in leaves and ovaries at SE, P, and 3DAP, and in silks at SE and P. In leaves, sucrose and starch are the major reserve forms of carbohydrates. The sucrose and starch contents in leaves of the W group were similar to, or higher than, those in CK, but their contents were significantly decreased in leaves of the S and WS groups from P onward (Figures 3A–D). The leaf sucrose and starch contents were significantly lower in the WS group than in the W or S groups from P onward. For ovaries, the sugar and starch contents remained stable or increased in the W group (Figures 3E–H). The ovary sugar and starch contents were generally lower in the S and WS groups than in CK from P, except for the sucrose content in ovaries of the S group at 3DAP. Moreover, the ovary sugar and starch contents were significantly lower in the WS group than in the W or S groups from P. For silks, compared with CK, the W group showed significantly higher sucrose and starch contents, and similar fructose and glucose contents (Figures 3I–L). For the S and WS groups, the sugar and starch contents in silks were significantly lower than those in CK from SE. Additionally, the sugar and starch contents in silks were significantly lower in the WS group than in the W and S groups. In general, the sugar and starch contents in source and sink tissues were maintained or increased in the W group, but decreased in the S and WS groups, and were lowest in the WS group.

**FIGURE 4:**
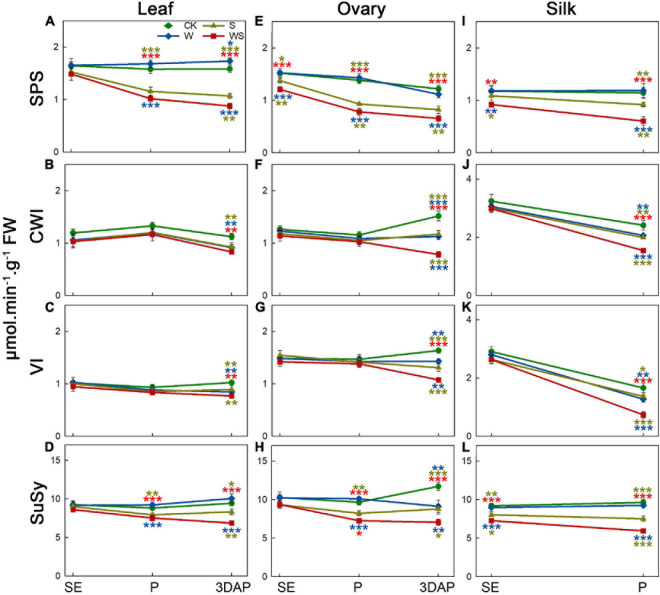
Enzyme activities in leaf, ovary, and silks. Activities of sucrose phosphate synthase (SPS), cell wall invertase (CWI), vacuolar invertase (VI), and sucrose synthase (SuSy) in maize leaf **(A–D)** and ovary **(E–H)** at SE (first silk emergence), P (full silk emergence), and 3DAP (3 days after pollination). Activities of SPS **(I)**, CWI **(J)**, VI **(K)**, and SuSy **(L)** in silks at SE and P. Colored asterisks above indicate significant differences between control (CK) and treatment groups (waterlogging + shading, WS; waterlogging, W; shading, S). Colored asterisks below indicate significant differences between WS and W or S groups. Error bars represent ± SD. (*n* = 3). *P < 0.05, **P < 0.01, ***P < 0.001 (ANOVA and *t*-test).

To assess the effect of the treatments on carbohydrate metabolic enzymes, we determined the activities of SPS, CWI, VI, and SuSy in leaves, ovaries, and silks. For leaves, the SPS and SuSy activities in the W group remained stable or were higher than those in CK, while CWI and VI activities also remained stable until P and decreased at 3DAP (Figures 4A–D). The SPS and SuSy activities in leaves were lower in the S and WS groups than in CK from P, and the CWI and VI activities in leaves were decreased at 3DAP. The SPS and SuSy activities in leaves were significantly lower in the WS group than in the W and S groups. For ovaries, SPS and SuSy activities remained stable in the W group, except that SuSy activity was decreased at 3DAP (Figures 4E,H). In the S and WS groups, the SPS activity in ovaries was lower than that in CK from SE, and the SuSy activity decreased from P. At 3DAP, the activities of CWI and VI in ovaries were decreased in all treatment groups, compared with CK, and were lowest in the WS group (Figures 4F,G). For silks, SPS and SuSy activities remained stable in the W group (Figures 4I,L). In the S group, the SPS activity in silks decreased at P. In silks of the WS group, SPS activity was lower than that in CK from SE. The activity of SuSy from SE was lower in the S and WS groups than in CK, and the activities of SPS and SuSy were significantly lower in the WS group than in the W or S groups. The CWI and VI activities in silks were maintained at SE but decreased at P in all treatment groups, compared with CK, and were lowest in the WS group (Figures 4J,K). Overall, SPS and SuSy activities remained stable or were higher in the W group and lower in the S and WS groups than in CK, and the CWI and VI activities in leaves and ovaries were decreased in all the treatment groups at 3DAP, with the lowest activities in the WS group.

### Quasi-Targeted Metabolomics Analyses of Leaves, Ovaries, and Silks Among Waterlogging, Shading, and Combined Waterlogging and Shading Groups

**FIGURE 5:**
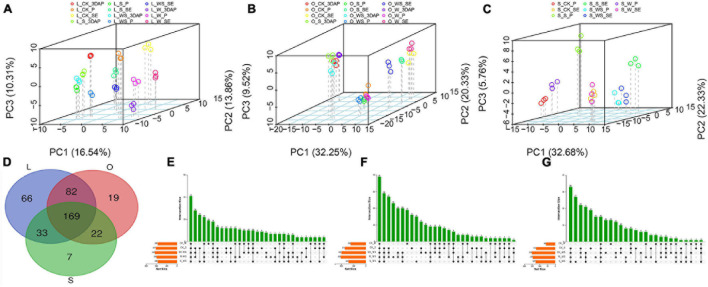
Principal component analyses (PCA) and Venn diagrams showing results of metabolomics analyses. PCA of metabolites in leaves **(A)**, ovaries **(B)**, and silks **(C)**. Venn diagrams showing overlapping of differentially accumulated metabolites in different tissues **(D)** and overlapping of differentially accumulated metabolites in leaves **(E)**, ovaries **(F)**, and silks **(G)** in control (CK) and waterlogging + shading (WS), waterlogging (W), and shading (S) treatment groups. L (leaf), O (ovary), S (silk).

To further analyze the metabolic changes in response to the W, S, and WS treatments, quasi-targeted metabolomics analyses were performed for maize leaves, ovaries, and silks. A total of 430 metabolites were detected in leaves, ovaries, and silks (Supplementary File 2). PCA were performed for metabolites in all tissues and each tissue (Figures 5A–C and Supplementary Figure 4). The results confirmed a high level of repeatability and revealed tissue-specific accumulation patterns of metabolites. Based on the KEGG analyses, the 430 metabolites were mapped to 13 pathways, most of which were related to organic acids, amino acids, carbohydrates, and nucleotides (Supplementary Figure 5).

In total, 350, 292, and 231 DAMs were detected for all treatments in leaves, ovaries, and silks, respectively (Figure 5D). Of them, 169 DAMs overlapped among the three tissues, while 66, 19, and 7 accumulated specifically in leaves, ovaries, and silks, respectively. In leaves, 189, 207, and 216 DAMs were identified in the W, S, and WS groups, respectively, compared with CK (Figure 5E); and 198 and 189 DAMs were identified in the W and S groups, respectively, compared with the WS group. In ovaries, 124, 171, and 199 DAMs were identified in the W, S, and WS groups, respectively, compared with CK (Figure 5F); and 203 and 141 DAMs were identified in the W and S groups, respectively, compared with the WS group. In silks, 60, 124, and 153 DAMs were identified in the W, S, and WS groups, respectively, compared with CK (Figure 5G); and 145 and 127 DAMs were identified in the W and S groups, respectively, compared with the WS group. Therefore, the patterns of metabolite accumulation differed among leaves, ovaries, and silks. The largest number of DAMs was in the WS group, consistent with the more serious negative effects of the WS treatment than the W or S treatments alone.

### Identification of Metabolites Closely Related to Ovarian Abortion by Weighted Gene Co-expression Network Analysis of Leaves, Ovaries, and Silks by Waterlogging, Shading, and Combined Waterlogging and Shading Treatments

To investigate which metabolites were related to agronomic and physiological traits under the different treatments during the flowering stage, metabolic data were subjected to WGCNA. The 430 metabolites were assigned to six co-expression modules with different colors (yellow, blue, green, turquoise, brown, and red) (Supplementary Figure 6 and Supplementary File 2).

**FIGURE 6:**
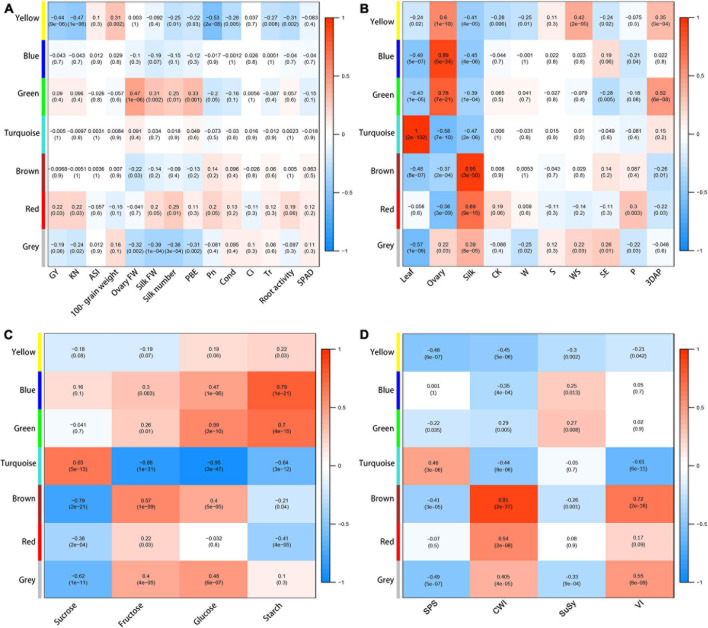
Heat map and Pearson’s correlations with between modules and agronomic and physiological traits **(A)**, organs or treatments **(B)**, sugar contents **(C)**, and enzymatic activity **(D)**. Gray modules represent metabolites that cannot be categorized.

To explore the potential functions of each module, we determined correlations between each module and yield and physiological changes. The yellow module was negatively correlated with GY (−0.44), KN (−0.47), *P*_*n*_ (−0.53), silk number (−0.25), PBE (−0.22), cond. (−0.28), *T*_*r*_ (−0.27), and root activity (−0.31), and positively correlated with ASI (0.31) (Figure 6A). The red module was positively correlated with GY (0.22), KN (0.22), and silk number (0.25). The results showed that the yellow, blue, and green modules had strong positive correlations with ovary parameters; the turquoise module had a strong positive correlation with leaf parameters; and the brown and red modules had strong positive correlations with silk parameters (Figure 6B), indicating that the different modules represented special expression patterns in ovaries, silks, and ovaries. The blue, green, turquoise, and brown modules had no significant correlations with yield, but they showed strong correlations with some other physiological traits (Figure 6). We detected strong positive correlations between the blue module and starch content (0.79); and between the green module and ovary FW (0.47) and glucose content (0.59). The turquoise module had a strong positive correlation with sucrose content (0.65), and negative correlations with fructose content (−0.88), glucose content (−0.95), and VI activity (−0.61). The brown module had positive correlations with fructose content (0.57), CWI activity (0.91), and VI activity (0.72), and a negative correlation with sucrose content (−0.79).

Next, eigengene expression analyses of the yellow and red module showed that the accumulation pattern of metabolites in these modules showed strong tissue-specific trends (Supplementary Figure 7). The metabolites in the yellow module showed increased contents in the ovaries in the treatment groups compared with CK, with the highest relative contents in the WS group (Supplementary Figure 7A). The metabolites in the red module showed lower contents in the silks of treatment groups, especially WS, than in silks of CK (Supplementary Figure 7B). This accumulation pattern was most pronounced at P. The yellow module contained 27 metabolites, most of which were amino acids and their derivatives including BCAAs (leucine, isoleucine, and valine), tryptophan, histidine, and alanine (Supplementary File 2). The red module contained 11 metabolites, mainly flavonoids, include quercetin and diosmetin.

**FIGURE 7:**
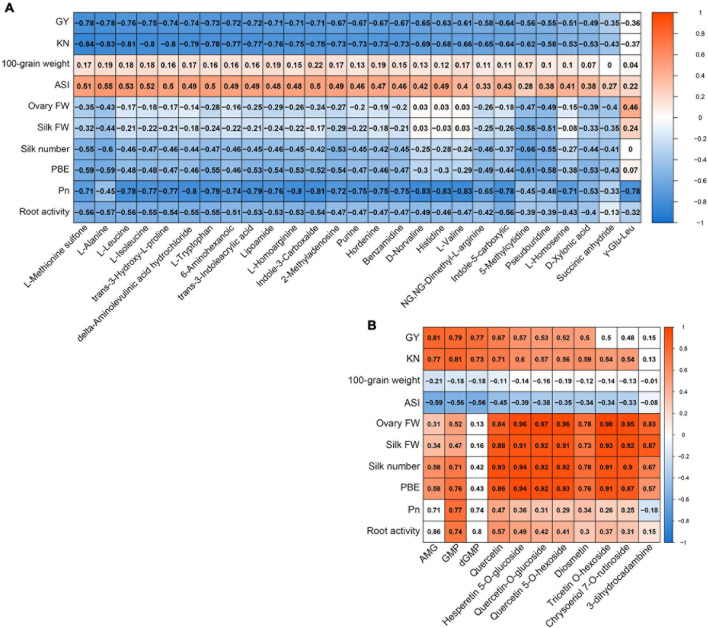
Correlation coefficient matrix of metabolites from yellow module whose accumulation patterns in ovary correlate with agronomic and source strength traits **(A)**. Correlation coefficient matrix of metabolites from red module whose accumulation patterns in silk correlate with agronomic and source strength traits **(B)**. Blue fill color, significant negative correlation; red fill color, significant positive correlation (*P* < 0.05); white fill color, *P* ≥ 0.05.

We conducted correlation analyses to determine whether the metabolites from the yellow module in ovaries or from the red module in silks were related to any of the main agronomic and physiological traits (Figure 7). The majority of metabolites from the yellow module in ovaries had strong negative correlations with GY (< −0.6), KN (< −0.65), *P*_*n*_ (< −0.7), and root activity (< −0.5), but positive correlations with ASI (> 0.45) (Figure 7A). The majority of metabolites from the red module in silks showed strong positive correlations with GY (>0.5), KN (>0.5), ovary FW (>0.8), silk FW (>0.8), silk number (>0.8), and PBE (>0.8) (Figure 7B). In conclusion, the accumulation patterns of metabolites in the yellow module in ovaries were strong positively correlated with ovary abortion, while the accumulation patterns of metabolites in the red module in silks were strongly negatively correlated with ovary abortion.

### Effects of Waterlogging, Shading, and Combined Waterlogging and Shading Treatments on Amino Acid and Carbohydrate Metabolic Pathways

The W, S, and WS treatments had different effects on amino acid and carbohydrate metabolism in leaves, ovaries, and silks. The affected pathways included glycolysis, pentose phosphate pathway, the TCA cycle, glutathione metabolism, urea cycle, and the MET salvage pathway.

In leaves, the effects of the WS and S treatments on glycolysis intermediate metabolites were different from the effects of the W treatment. Specifically, 3-phosphoglycerate and phosphoglycerate (PEP) were significantly less abundant in the W group than in CK, while PEP and fructose-1,6-P (fructose-1,6-phosphate) showed increasing trends in the S and WS groups, and the pyruvate content decreased in S and WS groups but remained stable in the W group (Supplementary Figure 8). Compared with the W treatment, the S treatment had the opposite effect on the pentose phosphate pathway. For example, the contents of ribulose-5P (ribulose-5-phosphate), ribose-5P (ribose-5-phosphate), and glyceraldehyde-3P (glyceraldehyde-3-phosphate) were decreased in the W group and increased in the S group. The TCA cycle was differently affected by the W, S, and WS treatments. Compared with CK, the W treatment resulted in decreased contents of malate, fumarate and α-ketoglutarate at SE, increased fumarate content at P, and decreased succinate content at 3DAP. Compared with CK, the S treatment resulted in decreased isoleucine content at SE; and the WS treatment resulted in decreased isoleucine, α-ketoglutarate, and succinate contents at SE, but increased α-ketoglutarate, fumarate, and malate contents at 3DAP. In terms of glutathione metabolism, the contents of glutamate and 5-oxoproline were decreased in the W group, but almost unaffected in the S and WS groups. The accumulation of citrulline in the urea cycle was significantly down-regulated in all treatment groups, the asparagine and ornithine were down-regulated in W and S groups but up-regulated in the WS group. The MET salvage pathway intermediates were almost unaffected by the three treatments. In addition, the three stress treatments significantly affected multiple amino acids related to these metabolic pathways, especially BCAAs, whose biosynthesis and degradation are related to glycolysis and the TCA cycle. The contents of BCAAs were increased in the S and WS groups at SE and P, but decreased at 3DAP in the W and S groups. All stress treatments resulted in decreased contents of serine, alanine, and glutamine. In general, our treatments changed the metabolic networks in leaves. The WS and S treatments had similar effects on many metabolites, but the effects of the W treatment were different.

**FIGURE 8:**
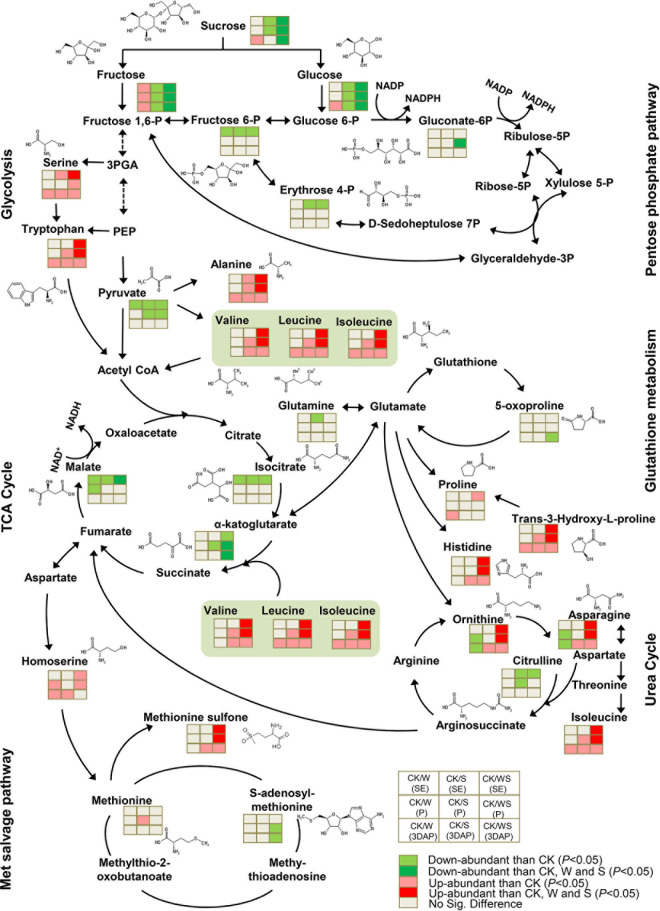
Differences in metabolites involved in carbohydrate and amino acid metabolism in ovaries affected by waterlogging + shading (WS), waterlogging (W), and shading (S) treatment at SE (first silk emergence), P (full silk emergence) and 3DAP (3 days after pollination). Grids next to each metabolite represent accumulation of corresponding metabolites in the W, S, and WS groups at each time point. Colors correspond to the significance of the change in accumulation. Light red: more abundant than in control (*P* < 0.05). Deep red: more abundant than in control, W, and S (*P* < 0.05). Light green: less abundant than in control (*P* < 0.05). Deep green: less abundant than in control, W, and S (*P* < 0.05). Light gray: no significant difference compared with control.

In ovaries, the stress treatments had little effect on intermediate metabolites in glycolysis and the pentose phosphate pathways, but all treatments down-regulated pyruvate (Figure 8). In the TCA cycle, isoleucine, α-ketoglutarate, and malate were significantly down-regulated in all treatment groups, and α-ketoglutarate and malate accumulated to significantly lower levels in the WS group than in the W and S groups. Although the treatments had limited effects on glutathione metabolism, 5-oxoproline was down-regulated in the WS group. In the urea cycle, ornithine and asparagine were down-regulated in the W group but up-regulated in the S and WS groups, and to significantly higher levels in the WS group than in the W and S groups. Citrulline was down-regulated in the S and WS groups but remained at a constant level in the W group. In the MET salvage pathway, the *S*-adenosylmethionine content was down-regulated in the WS group, but not affected in the W and S groups. Likewise, most amino acids related to glycolysis and the TCA cycle were up-regulated in all treatment groups. These amino acids, which included BCAAs, tryptophan, arginine, and alanine, accumulated to significantly higher levels in the WS group than in the W and S groups. All the treatments affected the metabolic network in the ovary by decreasing the contents of TCA cycle intermediates and increasing the contents of some amino acids. These effects were most severe in the WS group.

In silks, glycolysis and the pentose phosphate pathway, fructose-1,6-P, glucose-6-P (glucose-6-phosphate), PEP, and erythrose-4-P (erythrose-4-phosphate) were up-regulated in the S and WS groups. Both PEP and erythrose-4-P accumulated to higher levels in the WS group than in the W and S groups (Supplementary Figure 9). In terms of components of the TCA cycle, compared with CK, all treatment groups showed decreased contents of α-ketoglutarate; the S and WS groups showed decreased contents of isocitrate and increased contents of fumarate; and the malate content was decreased in the W group but increased in the S group. Glutamate, which is involved in glutathione metabolism, was down-regulated in the S group. In the urea cycle, citrulline was up-regulated in the W group but down-regulated in the S and WS groups, and ornithine and asparagine were up-regulated in the WS group. In the MET salvage pathway, MET was up-regulated in the S and WS groups, and *S*-adenosylmethionine was down-regulated in the WS group. In addition, the related amino acids were up-regulated in the S and WS groups. In particular, some amino acids, including BCAAs, tryptophan, histidine, and MET sulfone, accumulated to higher levels in the WS group than in the W and S groups. The W treatment had little effect on these metabolic pathways in silks, but the effects of the S and WS treatments on amino acid metabolism in silks were similar to those in ovaries.

### Effect of Waterlogging, Shading, and Combined Waterlogging and Shading Treatments on H_2_O_2_ Content

**FIGURE 9:**
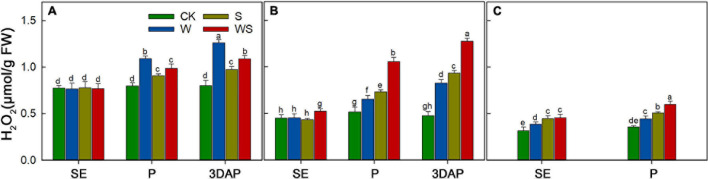
H_2_O_2_ contents in leaf **(A)**, ovary **(B)**, and silks **(C)** at SE (first silk emergence), P (full silk emergence), and 3DAP (3 days after pollination) in control (CK), waterlogging + shading (WS), waterlogging (W), and shading (S) treatment groups. Data were subjected to one-way ANOVA followed by Duncan’s new multiple range test (*n* = 3). Different letters above bars indicate significant differences (*P* < 0.05). Error bars represent ± SD.

To explore the effect of WS on maize antioxidant capacity, the H_2_O_2_ content was measured in leaves, ovaries, and silks. The results showed that H_2_O_2_ accumulated during the stress treatments in the W, S, and WS groups (Figure 9). At 3DAP, the H_2_O_2_ content in the W, S, and WS groups was 57.36%, 21.29%, and 36.8% higher than that in CK, respectively (Figure 9A). In ovaries, the H_2_O_2_ content was 74.23%, 96.14%, and 168.28% higher than that in CK, respectively (Figure 9B). At P, the H_2_O_2_ content in the silks in the W, S, and WS groups was 24.69%, 42.6%, and 67.98% higher than that in CK, respectively (Figure 9C). In conclusion, compared with the W or S groups, the WS group had higher H_2_O_2_ contents in ovaries and silks. The H_2_O_2_ content in leaves was lower in the WS group than in the W group.

## Discussion

In the North China Plain, combined waterlogging and shading severely affect maize yield, especially when these conditions occur around the flowering stage. However, most previous studies have focused on single waterlogging or shading stresses. Our results show that WS during flowering had stronger negative effects than did either stress alone, leading to greater ovary abortion. The observed ovary abortion was caused by the growth inhibition of ovaries and silks at the ear tip, which decreased the number of emerging silks, but this was independent of the carbon status. Our results also show that ovary abortion was closely related to specific amino acids and flavonoid metabolites in the ovaries and silks that are involved in energy metabolism and antioxidant capacity.

### Compared With Waterlogging and Shading, Combined Waterlogging and Shading More Severely Inhibited Ovary and Silk Growth at the Ear Tip, Resulting in a Higher Ovary Abortion Rate

Consistent with previous studies on the effects of waterlogging and shading (Ren et al., 2016a, b; Gao et al., 2017, 2020), all the treatments in our study decreased the photosynthetic rate and root activity of maize, and these effects were stronger in the WS group than in the W and S groups (Figure 1). Unlike other crops, such as wheat and soybean, maize shows non-constant biomass partitioning to the ear (Miralles et al., 1998; Rotundo et al., 2012). In a previous study, when the biomass of maize was decreased, the range of the decrease in PBE was much larger than the range of the decrease in plant biomass, and this characteristic was related to ovary abortion (Borrás and Vitantonio-Mazzini, 2018). In our study, therefore, the PBE was not just significantly lower in all the treatment groups than in CK (Figure 2E), but was lower in the WS group than in the W and S groups. This indicated that the WS treatment more seriously inhibited ear growth than did the W or S treatments. Thus, compared with the W and S treatments, the WS treatment had a greater impact on the source intensity and biomass allocation capacity of maize.

Previous studies have shown that the silks emerge from the ovaries at the base of the ear first, and from those at the tip of the ear last (Oury et al., 2016b), and that there is lower availability of assimilates to ovaries at the ear tip than at the ear base (Shen et al., 2018). Drought stress seriously affected the silk elongation of maize, which resulted silks at the ear tip or all locations could not extended from the bracts (Oury et al., 2016b; Danilevskaya et al., 2019). In all treatment groups in our study, the growth of ovaries and silks located the ear tip was strongly inhibited, and the aborted ovaries were concentrated at the ear tip (Figure 2). In addition, the final number of emerged silks was highly consistent with KN (Supplementary Figure 3). This indicated that in the W, S, and WS groups, many silks located at the ear tip failed to extend from the bracts, thus, many apical ovaries were not fertilized at pollination in the treatment groups. Therefore, W, S, and WS treatments at the flowering stage inhibited the growth of ovaries and silks at the tip ear, reduced the number of emerged silks, and resulted in a large number of ovaries remaining unfertilized at the ear tip, leading to abortion. This situation was more serious in the WS group than in the W and S groups.

### Changes in Carbon Status Caused by Waterlogging, Shading, and Combined Waterlogging and Shading Treatments During Flowering Could Not Explain Ovary Abortion

In our study, ovary abortion occurred whether the sugar content in leaves, ovaries, and silks decreased, stayed the same, or increased in the treatment groups. The sugar content decreased in the S group (Figure 3), consistent with previous studies (Liang et al., 2020; Wu et al., 2021). Few previous studies have investigated changes in sugar contents in maize under waterlogging or combined waterlogging and shading conditions during the flowering period. In our study, the sugar content was maintained or increased in the W treatment and decreased in the S and WS treatments, and the lowest sugar level was in the WS group. This suggested that the effect of WS on sugar content was mainly because of shading, but the decrease in sugar content was exacerbated by waterlogging. Although a previous study showed that sucrose feeding reverses kernel losses under shading stress, this effect is due to increased kernel weight, not increased KN (Hiyane et al., 2010). Like previous studies on the effects of drought or salt stress around the flowering time (Henry et al., 2015; Cagnola et al., 2018), we found that the W treatment resulted in ovary abortion even though the sugar status was maintained or increased.

Previous studies have obtained inconsistent results in terms of the correlation between invertase activity and ovary abortion in ovaries, and this may be related to the stage when treatments are applied. Changes in CWI activity under drought stress were found to be related to ovary abortion in some studies (Boyer, 2004; McLaughlin and Boyer, 2004; Chen et al., 2019), but their treatments began after silks emerged from the bracts and there were no data for silk growth. In another study, the CWI activity changed in both basal and apical ovaries of maize under a moderate drought treatment, but ovary abortion only occurred in apical ovaries (Oury et al., 2016a); that treatment began before silk emergence and decreased the number of emerged silks. The latest research suggests that the reason for ovary/kernel abortion under drought conditions varies depending on the phenological stage of development, i.e., whether the drought stress occurs at the pre- or postpollination phase (Turc and Tardieu, 2018; Shen et al., 2020). Moreover, a recent study on delayed pollination of maize ears showed that the abortion of the apical kernels under synchronous pollination conditions was not triggered by low CWI activity (Shen et al., 2018). In our study, although the CWI and VI activities decreased in ovaries at 3DAP (Figures 4F,G), many apical ovaries were not fertilized at pollination because of the decreased number of emerged silks. Therefore, the apical ovaries may already have been aborted at 3DAP, because the fertilization of basal (oldest) ovaries is sufficient to stop the development of apical (younger) ovaries and cause their abortion (Turc and Tardieu, 2018). The decrease of CWI and VI activities in ovaries at 3DAP may be the result of this event. Hence, we concluded that the changes in CWI and VI activities were not the cause of ovary abortion in the W, S and WS groups at the flowering stage.

In a previous study, ovary abortion related to carbon status occurred with a random distribution along the ear under drought stress, whether or not sucrose feeding was performed (McLaughlin and Boyer, 2004), but in our study, the aborted ovaries were concentrated at the ear tip (Figure 2M). As discussed above, we consider that the changes in carbon status could not explain ovary abortion in the W, S, and WS treatments at the flowering period.

### Combined Waterlogging and Shading Induced Changes in Carbohydrate Metabolism and Tricarboxylic Acid Cycle, Especially in Ovaries

Carbohydrate metabolism and the TCA cycle are important metabolic pathways that are affected by abiotic stress (Yang et al., 2018; Gao et al., 2020). The W, S, and WS treatments reduced the contents of several intermediates of glycolysis in ovaries (Figure 8), indicative of a reduced supply of these intermediates for amino acid metabolism. Gluconate-6P, a component of the pentose phosphate pathway, is critical for NADPH production. The amount of gluconate-6P in the ovaries was lower in the WS group than in the W and S groups, suggesting that the antioxidant capacity of the ovaries was more severely affected in the WS group than in the W and S groups. The TCA cycle is a hub that links carbohydrate, amino acid, and lipid metabolism, and several of its intermediates were less abundant in the ovaries in the W, S, and WS groups than in CK. The lowest levels were in the WS group, indicating that WS inhibited energy metabolism in ovaries more seriously than did each single stress. Together, these results show that, compared with the single stresses, WS more strongly inhibited energy metabolism and decreased the antioxidant capacity in ovaries, consistent with the growth status.

The S and WS treatments had similar effects on carbohydrate metabolism in silks, but some intermediate metabolites were more strongly affected by the WS treatment than the S treatment (Supplementary Figure 9). The abundance of gluconate-6P only decreased in the WS group, suggesting that the antioxidant capacity of silks was more severely affected by the WS treatment than by the single stress treatments. Waterlogging alone had little effect on carbohydrate metabolism in silks, but it inhibited energy metabolism. The TCA cycle in silks may not have been seriously inhibited by the S and WS treatments because of complementary changes in metabolite contents.

In leaves, the effects of S and WS on glycolysis and the pentose phosphate pathway were opposite to those of W, and some intermediate metabolites were up-regulated in the S and WS groups but down-regulated in the W group (Supplementary Figure 8). The W and WS treatments led to complementary changes in the contents of different metabolites in the TCA cycle in leaves, while the S treatment only led to the down-regulation of isoleucine at SE. These results indicate that the TCA cycle of leaves was less affected by these treatments.

### Effect of Combined Waterlogging and Shading on Amino Acid Metabolism in Ovaries Was Related to Abortion

Amino acid metabolism plays an important role in defense, signaling, and other processes. Compared with single stresses, WS stress more severely affected ovary amino acid metabolism. The biosynthesis and degradation of BCAAs, serine, tryptophan, and alanine are related to glycolysis or the TCA cycle. These compounds accumulated in the ovary in the W, S, and WS groups, and to the highest levels in the WS group (Figure 8). In plants, the levels of free amino acids, including BCAAs, increase in response to various abiotic stresses *via* either *de novo* synthesis or protein degradation (Galili et al., 2016; Batista Silva et al., 2019). Several studies have shown that, although the proline content increases in response to stress, there are often larger increases in the contents of other amino acids, especially BCAAs (Joshi et al., 2010; Sun et al., 2016; Huang and Jander, 2017). Extensive BCAAs accumulation in response to abiotic stress has been observed in rice, maize, and Arabidopsis (Virlouvet et al., 2011; Huang and Jander, 2017; Maksym et al., 2018; Sun et al., 2020). Alanine and tryptophan levels have been shown to increase under stresses (Pavlík et al., 2010a, b; Fu et al., 2018; Wang et al., 2019), an increased alanine level may be an indicator of unbalanced nitrogen nutrition (Atanasova, 2008). Therefore, many plants respond to abiotic stress by increasing the levels of these amino acids, especially BCAAs. Among all the treatments in this study, the WS treatment had the strongest effect on amino acid contents in the ovaries. However, the excessive accumulation of these amino acids may be detrimental to plant growth and development. Huang and Jander (2017) found that protein degradation led to the accumulation of BCAAs in *Arabidopsis* under drought stress. *ZmASR1*-overexpressing maize plants showed increased yields and KN under drought stress, and this may have been related to the decreased BCAAs contents and altered transcript levels of BCAAs-related genes (Virlouvet et al., 2011). Therefore, in our study, the stronger inhibition of energy metabolism in the ovary in the WS group may be related to the overaccumulation of these amino acids, especially BCAAs.

Components of glutathione metabolism such as 5-oxoproline showed decreased levels in the ovary in the WS group, suggesting that this treatment impaired the ability to repair oxidative damage. *Trans*-3-hydroxy-l-proline is an intermediate of proline metabolism that is present in the plant cell wall. In animal cells, this compound can scavenge ROS (Hu et al., 2021). In this study, its accumulation pattern was the same as that of BCAAs. The C/N balance is important to sustain the optimal growth and development of plants (Zheng, 2009; Zhang et al., 2019; Dong et al., 2020). The urea cycle plays an important role for maintaining the C/N balance. Compared with the S and WS treatments, the W treatment had a stronger effect on the urea cycle. This is because the decrease in citrulline in the S and WS groups was offset by the accumulation of ornithine and asparagine. This suggested that the C/N balance was better maintained in the S and WS groups than in the W group. Furthermore, this suggests that S treatment can mitigate the effects of W treatment in terms of the C/N balance in ovaries. The MET salvage pathway plays an important role in plant stress response. An intermediate of this pathway, *S*-adenosyl-methionine (SAM), can improve the tolerance of *Arabidopsis* to abiotic stress and H_2_O_2_ (Ezaki et al., 2016; Ma et al., 2017). In this study, the SAM levels in the ovary declined in the WS group, indicative of a decrease in tolerance. Protein-bound MET and free MET can be oxidized by ROS into MET sulfone (Simonović and Anderson, 2007). MET sulfone accumulated in the S and WS groups, and to higher levels in the WS group than in the W and S groups. This indicated that ROS accumulated to the highest levels in WS group ovaries, consistent with the H_2_O_2_ content (Figure 9B). Furthermore, the WGCNA analysis showed that the accumulation pattern of these amino acids (BCAAs, tryptophan, alanine, histidine, *trans*-3-hydroxy-L-proline, and MET sulfone) in ovaries was strongly positively correlated with ovary abortion. In summary, compared with single stresses, WS stress results in more energy allocated to the synthesis of stress-related amino acids in the ovary, but this leads to decreased energy metabolism and a decreased antioxidant capacity, overaccumulation of H_2_O_2_, and ultimately, ovary abortion.

Amino acid metabolism in silks and ovaries was similarly affected by the S and WS treatments, but less affected by the W treatment (Supplementary Figure 9). The effects on amino acid metabolism in leaves were different from those in ovaries. Compared with the S treatment, the WS treatment did not cause greater accumulation on amino acids in leaves, while the W treatment resulted in down-regulation of amino acids in leaves. The contents of these amino acids, especially glutamate and 5-oxoproline metabolized from glutathione, were decreased in the W group but maintained in the S and WS groups. This not only indicated that the W treatment might have decreased the antioxidant capacity of leaves, but also suggested that the S treatment could alleviate this decline, consistent with the H_2_O_2_ content (Figure 9A).

### Relationship Between Ovary Abortion and Decreased Contents of Some Flavonoids in Silks in Combined Waterlogging and Shading Treatment

Flavonoids exhibit diverse biological functions and they control key steps in cell differentiation and growth, but their metabolic pathways are unclear. Despite the controversy, Agati et al. (2012, 2020) think they are involved in the regulation of development at the tissue scale and the whole-plant scale, and this is attributed to their antioxidant properties. The flavonoid quercetin is known to affect plant growth during cold acclimation, and was shown to regulate primary root growth in rice (Fritz et al., 2007; Xu et al., 2019). Quercetin functions as an antioxidant to scavenge ROS. Some studies have detected an inverse relationship between flavonol content and ROS content (Watkins et al., 2017; Peng et al., 2019). Diosmetin is also an important antioxidant in the response to environmental stresses (Bazghaleh et al., 2018; Xie et al., 2021). The WGCNA analysis showed that the decreased contents of 11 specific metabolites (mainly flavonoids including quercetin and diosmetin) in silks was highly positively correlated with KN, silk FW, and silk number (Figure 8B). Their accumulation patterns in silks were similar to the changes in H_2_O_2_ content (Supplementary Figure 7B and Figure 9C). Our results indicate that the decreased contents of these flavonoids affected KN by inhibiting silk growth, and this may be related to their antioxidant properties. Thus, flavonoids metabolites may play an important role in maintaining the growth of silks in maize under abiotic stress.

This is the first report on the key phenotypic, physiological, and metabolic characteristics of WS at the flowering stage leading to ovary abortion. One of the limitations of this study is that we focused on the effects of the W, S, and WS stresses at the flowering stage. Further studies are required to explore the responses of maize plants at other developmental stages to combined stresses. Another limitation is that the role of these metabolites in relation to ovary abortion has not been verified by genome editing methods.

## Conclusion

Analyses of agronomic and physiological characteristics and quasi-targeted metabolomics analyses of maize leaves, ovaries, and silks have provided novel insights into the causes of ovary abortion in maize plants subjected to W, S, and WS treatments during the flowering period. Compared with W or S stresses alone, the WS treatment had more serious effects on the growth and reproduction of maize, resulting in a reduced source capacity, and increased ovary abortion. Compared with the single stresses, WS increased ovary abortion. This was not due to the change in carbon status, but was related to three factors: (1) a decrease in the number of emergent silks; (2) excessive resources diverted into the biosynthesis of stress-related amino acids in ovaries, leading to decreased energy metabolism and a lower antioxidant capacity that led to overaccumulation of H_2_O_2_; (3) decreased concentrations of specific flavonoids with antioxidant properties in silks, resulting in H_2_O_2_ accumulation.

The results of this study provide the basis for further research on how maize responds to W, S, and WS stresses during flowering. Our results also identify target traits for breeding, such as silk growth status, amino acids and flavonoid metabolite contents in ovaries and silks, and antioxidant capacity. In addition, the metabolites closely related to abortion, such as BCAAs, alanine, quercetin, diosmetin, and other metabolites, are potential targets for genome editing and other biotechnological strategies to improve maize yield.

## Data Availability Statement

The raw data supporting the conclusions of this article will be made available by the authors, without undue reservation.

## Author Contributions

JZ designed and conducted the experiment, performed data analysis, and drafted the manuscript. LT performed data analysis and manuscript revision. SW and HL helped draft the manuscript. YZ, MZ, XW, and PA assisted in the execution of the experiment. CL conceived and coordinated the study. All authors read the manuscript and approved the submission.

## Conflict of Interest

The authors declare that the research was conducted in the absence of any commercial or financial relationships that could be construed as a potential conflict of interest.

## Publisher’s Note

All claims expressed in this article are solely those of the authors and do not necessarily represent those of their affiliated organizations, or those of the publisher, the editors and the reviewers. Any product that may be evaluated in this article, or claim that may be made by its manufacturer, is not guaranteed or endorsed by the publisher.

## Abbreviations

CK: control
W: waterlogging stress
S: shading stress
WS: combined waterlogging and shading stress
SE: first silk emergence
P: full silk emergence
3DAP: 3 days after pollination
ASI: anthesis-silking interval
GY: grain yield
TCA cycle: tricarboxylic acid cycle
PBE: proportion of biomass allocated to ears
*P*_*n*_: net photosynthetic rate
*G*_*s*_: stomatal conductance
*T*_*r*_: transpiration rate
*C*_*i*_: intercellular CO_2_ concentration
SPS: sucrose phosphate synthase
CWI: cell wall invertase
VI: vacuolar invertase
SuSy: sucrose synthase
H_2_O_2_: hydrogen peroxide
ROS: reactive oxygen species
WGCNA: weighted gene co-expression network analysis
FW: fresh weight
DW: dry weight
KN: kernel number
PCA: principal component analyses
KEGG: Kyoto Encyclopedia of Genes and Genomes
DAMs: differentially accumulated metabolites
BCAAs: branched-chain amino acids
MET: methionine.

## References

[B1] Agati G. Azzarello E. Pollastri S. Tattini M. (2012). Flavonoids as antioxidants in plants: location and functional significance. *Plant Sci.* 196 67–76. 10.1016/j.plantsci.2012.07.014 23017900

[B2] Agati G. Brunetti C. Fini A. Gori A. Guidi L. Landi M. (2020). Are flavonoids effective antioxidants in plants? *Antioxidants* 9:1098. 10.3390/antiox9111098 33182252 PMC7695271

[B3] An P.-P. Ming B. Dong P. F. Zhang M. Li C.-H. (2018). Response of maize (*Zea mays* L.) yield to climatic ecological condition on the south Yellow-Huaihe-Haihe rivers plain. *Acta Agrono. Sin*. 44 442–453. 10.3724/SP.J.1006.2018.00442

[B4] Atanasova E. (2008). Effect of nitrogen sources on the nitrogenous forms and accumulation of amino acid in head cabbage. *Plant Soil Environ.* 54 66–71. 10.17221/438-PSE

[B5] Batista Silva W. Heinemann B. Rugen N. Nunes Nesi A. Araújo W. L. Braun H. P. (2019). The role of amino acid metabolism during abiotic stress release. *Plant Soil Environ.* 42 1630–1644. 10.1111/pce.13518 30632176

[B6] Bazghaleh N. Prashar P. Purves R. W. Vandenberg A. (2018). Polyphenolic composition of lentil roots in response to infection by *Aphanomyces euteiches*. *Front. Plant Sci.* 9:1131. 10.3389/fpls.2018.01131 30123232 PMC6085569

[B7] Bellasio C. Griffiths H. (2014). Acclimation of c4 metabolism to low light in mature maize leaves could limit energetic losses during progressive shading in a crop canopy. *J. Exp. Bot.* 65 3725–3736. 10.1093/jxb/eru052 24591058 PMC4085954

[B8] Bernardi J. Battaglia R. Bagnaresi P. Lucini L. Marocco A. (2019). Transcriptomic and metabolomic analysis of ZmYUC1 mutant reveals the role of auxin during early endosperm formation in maize. *Plant Sci.* 281 133–145. 10.1016/j.plantsci.2019.01.027 30824046

[B9] Borrás L. Vitantonio-Mazzini L. N. (2018). Maize reproductive development and kernel set under limited plant growth environments. *J. Exp. Bot.* 69 3235–3243. 10.1093/jxb/erx452 29304259

[B10] Boyer J. S. (2004). Grain yields with limited water. *J. Exp. Bot.* 55 2385–2394. 10.1093/jxb/erh219 15286147

[B11] Boyer J. S. (2010). Drought decision-making. *J. Exp. Bot.* 61 3493–3497. 10.1093/jxb/erq231 20667963

[B12] Cagnola J. I. Dumont, De Chassart G. J. Ibarra S. E. Chimenti C. Ricardi M. M. (2018). Reduced expression of selectedfasciclin-like arabinogalactan protein genes associates with the abortion of kernels in field crops ofzea mays (maize) and of arabidopsis seeds. *Plant Cell Environ.* 41 661–674. 10.1111/pce.13136 29314044

[B13] Chen L. Liu X. Huang X. Luo W. Long Y. (2019). Functional characterization of a drought-responsive invertase inhibitor from maize (Zea mays L.). *Int. J. Mol. Sci.* 20:4081. 10.3390/ijms20174081 31438536 PMC6747265

[B14] Chen Y. Chen X. Wang H. Bao Y. Zhang W. (2014). Examination of the leaf proteome during flooding stress and the induction of programmed cell death in maize. *Proteome Sci.* 12:33. 10.1186/1477-5956-12-33 25028572 PMC4099015

[B15] Cotrozzi L. Landi M. (2018). “*Molecular and physiological adaptations of tea plant in response to low light and UV stress*,” in *Stress Physiology of Tea in the Face of Climate Change*, eds Han W. Y. Li X. Ahammed G. J. (Singapore: Springer Singapore), 83–110.

[B16] Cui H. Camberato J. J. Jin L. Zhang J. (2015). Effects of shading on spike differentiation and grain yield formation of summer maize in the field. *Int. J. Biometeorol.* 59 1189–1200. 10.1007/s00484-014-0930-5 25380975

[B17] Cui H. Y. Zhang J. W. Jin L. B. (2012). Effects of shading on stalks morphology, structure and lodging of summer maize in field. *Sci. Agric. Sin.* 17 3497–3505.

[B18] Danilevskaya O. N. Yu G. Meng X. Xu J. Stephenson E. Estrada S. (2019). Developmental and transcriptional responses of maize to drought stress under field conditions. *Plant Direct* 3:e00129. 10.1002/pld3.129 31245774 PMC6589525

[B19] Dong X. Duan S. Wang H. B. Jin H. L. (2020). Plastid ribosomal protein LPE2 is involved in photosynthesis and the response to C/N balance in Arabidopsis thaliana. *J. Integr. Plant Biol.* 62 1418–1432. 31944575 10.1111/jipb.12907 PMC7540278

[B20] Ezaki B. Higashi A. Nanba N. Nishiuchi T. (2016). An s-adenosyl methionine synthetase (SAMS) gene from *Andropogon virginicus* L. Confers aluminum stress tolerance and facilitates epigenetic gene regulation in Arabidopsis thaliana. *Front. Plant Sci.* 7:1627. 10.3389/fpls.2016.01627 27877178 PMC5099669

[B21] Feng Y. H. Jiang Y. L. Xiao J. F. (2011). An experiment study on submergence tolerance of summer maize in east Henan plain. *J. Irrig. Drain.* 30 120–122.

[B22] Fritz D. Bernardi A. P. Haas J. S. Ascoli B. M. Bordignon S. A. D. L. von Poser G. L. (2007). Germination and growth inhibitory effects of hypericum myrianthum and H. *Revista Brasileira De Farmacognosia* 17 44–48. 10.1590/S0102-695X2007000100010

[B23] Fu H. Yu H. Li T. Zhang X. (2018). Influence of cadmium stress on root exudates of high cadmium accumulating rice line (Oryza sativa L.). *Ecotoxicol. Environ. Saf.* 150 168–175. 10.1016/j.ecoenv.2017.12.014 29276952

[B24] Galili G. Amir R. Fernie A. R. (2016). The regulation of essential amino acid synthesis and accumulation in plants. *Annu. Rev. Plant Biol.* 67 153–178. 10.1146/annurev-arplant-043015-112213 26735064

[B25] Gálvez Ranilla L. (2020). The application of metabolomics for the study of cereal corn (Zea mays L.). *Metabolites* 10:300. 10.3390/metabo10080300 32717792 PMC7463750

[B26] Gao J. Liu Z. Zhao B. Liu P. Zhang J. (2020). Physiological and comparative proteomic analysis provides new insights into the effects of shade stress in maize (Zea mays L.). *BMC Plant Biol.* 20:60. 10.1186/s12870-020-2264-2 32024458 PMC7003340

[B27] Gao J. Zhao B. Dong S. Liu P. Ren B. Zhang J. (2017). Response of summer maize photosynthate accumulation and distribution to shading stress assessed by using 13co2 stable isotope tracer in the field. *Front. Plant Sci.* 8:1821. 10.3389/fpls.2017.01821 29123536 PMC5662628

[B28] Gibon Y. Pyl E. T. Sulpice R. Lunn J. E. Höhne M. Günther M. (2009). Adjustment of growth, starch turnover, protein content and central metabolism to a decrease of the carbon supply when Arabidopsis is grown in very short photoperiods. *Plant Cell Environ.* 32 859–874. 10.1111/j.1365-3040.2009.01965.x 19236606

[B29] Hendriks J. H. M. Kolbe A. Gibon Y. Stitt M. Geigenberger P. (2003). ADP-glucose pyrophosphorylase is activated by posttranslational redox-modification in response to light and to sugars in leaves of Arabidopsis and other plant species. *Plant Physiol.* 133 838–849. 10.1104/pp.103.024513 12972664 PMC219057

[B30] Henry C. Bledsoe S. W. Griffiths C. A. Kollman A. Paul M. J. Sakr S. (2015). Differential role for trehalose metabolism in salt-stressed maize. *Plant Physiol.* 169 1072–1089. 10.1104/pp.15.00729 26269545 PMC4587459

[B31] Hiyane R. Hiyane S. Tang A. C. Boyer J. S. (2010). Sucrose feeding reverses shade-induced kernel losses in maize. *Ann. Bot.* 106 395–403. 10.1093/aob/mcq132 20616114 PMC2924829

[B32] Hu S. He W. Wu G. (2021). Hydroxyproline in animal metabolism, nutrition, and cell signaling. *Amino Acids* [Epub ahead of print]. 10.1007/s00726-021-03056-x 34342708

[B33] Huang T. Jander G. (2017). Abscisic acid-regulated protein degradation causes osmotic stress-induced accumulation of branched-chain amino acids in Arabidopsis thaliana. *Planta* 246 737–747. 10.1007/s00425-017-2727-3 28668976

[B34] Hütsch B. W. Saqib M. Osthushenrich T. Schubert S. (2014). Invertase activity limits grain yield of maize under salt stress. *J. Plant Nutr. Soil Sci.* 177 278–286. 10.1002/jpln.201300345

[B35] Jing F. U. Chao-Hai L. I. Zhao J. R. Liu T. X. (2009). Comparison of photosynthetic rate, grain yield and quality of different maize hybrids under low-light conditions. *J. Henan Agric. Univ.* 43 130–134.

[B36] Joshi V. Joung J. Fei Z. Jander G. (2010). Interdependence of threonine, methionine and isoleucine metabolism in plants: accumulation and transcriptional regulation under abiotic stress. *Amino Acids* 39 933–947. 10.1007/s00726-010-0505-7 20186554

[B37] Jung S. Hütsch B. W. Schubert S. (2017). Salt stress reduces kernel number of corn by inhibiting plasma membrane H^+^-ATPase activity. *Plant Physiol. Biochem.* 113 198–207. 10.1016/j.plaphy.2017.02.009 28236753

[B38] Kaur G. Vikal Y. Kaur L. Kalia A. Mittal A. Kaur D. (2021). Elucidating the morpho-physiological adaptations and molecular responses under long-term waterlogging stress in maize through gene expression analysis. *Plant Sci.* 304:110823. 10.1016/j.plantsci.2021.110823 33568312

[B39] Langfelder P. Horvath S. (2008). WGCNA: an R package for weighted correlation network analysis. *BMC Bioinformatics* 9:559. 10.1186/1471-2105-9-559 19114008 PMC2631488

[B40] Li Y. Tao H. Zhang B. Huang S. Wang P. (2018). Timing of water deficit limits maize kernel setting in association with changes in the source-flow-sink relationship. *Front. Plant Sci.* 9:1326. 10.3389/fpls.2018.01326 30405644 PMC6204571

[B41] Liang X. Gao Z. Shen S. Paul M. J. Zhang L. Zhao X. (2020). Differential ear growth of two maize varieties to shading in the field environment: effects on whole plant carbon allocation and sugar starvation response. *J. Plant Physiol.* 251:153194. 10.1016/j.jplph.2020.153194 32563766

[B42] Ma C. Wang Y. Gu D. Nan J. Chen S. Li H. (2017). Overexpression of s-Adenosyl-l-Methionine synthetase 2 from sugar beet m14 increased Arabidopsis tolerance to salt and oxidative stress. *Int. J. Mol. Sci.* 18:847. 10.3390/ijms18040847 28420190 PMC5412431

[B43] Maksym R. P. Ghirardo A. Zhang W. von Saint Paul V. Lange B. Geist B. (2018). The defense-related isoleucic acid differentially accumulates in arabidopsis among branched-chain amino acid-related 2-hydroxy carboxylic acids. *Front. Plant Sci.* 9:766. 10.3389/fpls.2018.00766 29937770 PMC6002512

[B44] McLaughlin J. E. Boyer J. S. (2004). Sugar-responsive gene expression, invertase activity, and senescence in aborting maize ovaries at low water potentials. *Ann. Bot.* 94 675–689. 10.1093/aob/mch193 15355866 PMC4242214

[B45] Miralles D. J. Katz S. D. Colloca A. Slafer G. A. (1998). Floret development in near isogenic wheat lines differing in plant height. *Field Crops Res.* 59 21–30.

[B46] Obata T. Witt S. Lisec J. Palacios-Rojas N. Florez-Sarasa I. Yousfi S. (2015). Metabolite profiles of maize leaves in drought, heat, and combined stress field trials reveal the relationship between metabolism and grain yield. *Plant Physiol.* 169 2665–2683. 10.1104/pp.15.01164 26424159 PMC4677906

[B47] Oury V. Tardieu F. Turc O. (2016b). Ovary apical abortion under water deficit is caused by changes in sequential development of ovaries and in silk growth rate in maize1[open]. *Plant Physiol.* 171 986–996. 10.1104/pp.15.00268 26598464 PMC4902573

[B48] Oury V. Caldeira C. F. Prodhomme D. Pichon J. Gibon Y. Tardieu F. (2016a). Is change in ovary carbon status a cause or a consequence of maize ovary abortion in water deficit during flowering? *Plant Physiol.* 171 997–1008. 10.1104/pp.15.01130 27208256 PMC4902574

[B49] Pavlík M. Pavlíková D. Balík J. Neuberg M. (2010a). The contents of amino acids and sterols in maize plants growing under different nitrogen conditions. *Plant Soil Environ.* 56 125–132. 10.17221/214/2009-PSE

[B50] Pavlík M. Pavlíková D. Staszková L. Neuberg M. Kaliszová R. Száková J. (2010b). The effect of arsenic contamination on amino acids metabolism in spinacia oleracea L. *Ecotoxicol. Environ. Saf.* 73 1309–1313. 10.1016/j.ecoenv.2010.07.008 20655589

[B51] Peng X. Wu H. Chen H. Zhang Y. Qiu D. Zhang Z. (2019). Transcriptome profiling reveals candidate flavonol-related genes of Tetrastigma hemsleyanum under cold stress. *BMC Genomics* 20:687. 10.1186/s12864-019-6045-y 31472675 PMC6717372

[B52] Ren B. Cui H. Camberato J. J. Dong S. Liu P. Zhao B. (2016a). Effects of shading on the photosynthetic characteristics and mesophyll cell ultrastructure of summer maize. *Naturwissenschaften* 103:67. 10.1007/s00114-016-1392-x 27437706

[B53] Ren B. Dong S. Zhao B. Liu P. Zhang J. (2017). Responses of nitrogen metabolism, uptake and translocation of maize to waterlogging at different growth stages. *Front. Plant Sci.* 8:1216. 10.3389/fpls.2017.01216 28744299 PMC5504228

[B54] Ren B. Hu J. Liu P. Zhao B. Zhang J. (2021). Responses of nitrogen efficiency and antioxidant system of summer maize to waterlogging stress under different tillage. *PeerJ* 9:e11834. 10.7717/peerj.11834 34395080 PMC8320525

[B55] Ren B. Zhang J. Dong S. Liu P. Zhao B. (2016b). Effects of waterlogging on leaf mesophyll cell ultrastructure and photosynthetic characteristics of summer maize. *PLoS One* 11:e0161424. 10.1371/journal.pone.0161424 27583803 PMC5008783

[B56] Rotundo J. L. Borrás L. Bruin J. D. Pedersen P. (2012). Physiological strategies for seed number determination in soybean: biomass accumulation, partitioning and seed set efficiency. *Field Crops Res.* 135 58–66. 10.1016/j.fcr.2012.06.012

[B57] Ruan Y. Llewellyn D. J. Furbank R. T. (2003). Suppression of sucrose synthase gene expression represses cotton fiber cell initiation, elongation, and seed development. *Plant Cell* 15 952–964. 10.1105/tpc.010108 12671090 PMC152341

[B58] Shen S. Liang X. Zhang L. Zhao X. Liu Y. Lin S. (2020). Intervening in sibling competition for assimilates by controlled pollination prevents seed abortion under postpollination drought in maize. *Plant Cell Environ.* 43 903–919. 10.1111/pce.13704 31851373

[B59] Shen S. Zhang L. Liang X. Zhao X. Lin S. Qu L. H. (2018). Delayed pollination and low availability of assimilates are major factors causing maize kernel abortion. *J. Exp. Bot.* 69 1599–1613. 10.1093/jxb/ery013 29365129 PMC5888920

[B60] Simonović A. D. Anderson M. D. (2007). Analysis of methionine oxides and nitrogen-transporting amino acids in chilled and acclimated maize seedlings. *Amino Acids* 33 607–613. 10.1007/s00726-007-0503-6 17334901

[B61] Sun C. X. Li M. Q. Gao X. X. Liu L. N. Wu X. F. Zhou J. H. (2016). Metabolic response of maize plants to multi-factorial abiotic stresses. *Plant Biol.* 18(Suppl. 1) 120–129. 10.1111/plb.12305 25622534

[B62] Sun Y. Shi Y. Liu G. Yao F. Zhang Y. Yang C. (2020). Natural variation in the osbzip18 promoter contributes to branched-chain amino acid levels in rice. *New Phytol.* 228 1548–1558. 10.1111/nph.16800 32654152

[B63] Turc O. Tardieu F. (2018). Drought affects abortion of reproductive organs by exacerbating developmentally driven processes via expansive growth and hydraulics. *J. Exp. Bot.* 69 3245–3254. 10.1093/jxb/ery078 29546424

[B64] Velikova V. Yordanov I. Edreva A. (2000). Oxidative stress and some antioxidant systems in acid rain-treated bean plants: protective role of exogenous polyamines. *Plant Sci.* 151 59–66. 10.1016/S0168-9452(99)00197-1

[B65] Virlouvet L. Jacquemot M. Gerentes D. Corti H. Bouton S. Gilard F. (2011). The ZmASR1 protein influences branched-chain amino acid biosynthesis and maintains kernel yield in maize under water-limited conditions. *Plant Physiol.* 157 917–936. 10.1104/pp.111.176818 21852416 PMC3192578

[B66] Wang L. Li X. Lian H. Ni D. He Y. Chen X. Y. (2010). Evidence that high activity of vacuolar invertase is required for cotton fiber and Arabidopsis root elongation through osmotic dependent and independent pathways, respectively. *Plant Physiol.* 154 744–756. 10.1104/pp.110.162487 20699399 PMC2948991

[B67] Wang R. Lin K. Chen H. Qi Z. Liu B. Cao F. (2021). Metabolome analysis revealed the mechanism of exogenous glutathione to alleviate cadmium stress in maize (Zea mays L.) Seedlings. *Plants* 10:105. 10.3390/plants10010105 33419127 PMC7825527

[B68] Wang X. Zenda T. Liu S. Liu G. Jin H. Dai L. (2019). Comparative proteomics and physiological analyses reveal important maize filling-kernel drought-responsive genes and metabolic pathways. *Int. J. Mol. Sci.* 20:3743. 10.3390/ijms20153743 31370198 PMC6696053

[B69] Wang Y. Tao H. Zhang P. Hou X. Sheng D. Tian B. (2020). Reduction in seed set upon exposure to high night temperature during flowering in maize. *Physiol. Plant.* 169 73–82. 10.1111/ppl.13049 31747055

[B70] Watkins J. M. Chapman J. M. Muday G. K. (2017). Abscisic acid-induced reactive oxygen species are modulated by Flavonols to control stomata aperture. *Plant Physiol.* 175 1807–1825. 10.1104/pp.17.01010 29051198 PMC5717730

[B71] Wen W. Li D. Li X. Gao Y. Li W. Li H. (2014). Metabolome-based genome-wide association study of maize kernel leads to novel biochemical insights. *Nat. Commun.* 5:3438. 10.1038/ncomms4438 24633423 PMC3959190

[B72] Wu H. Y. Tang H. K. Liu L. A. Shi L. Zhang W. F. Jiang C. D. (2021). Local weak light induces the improvement of photosynthesis in adjacent illuminated leaves in maize seedlings. *Physiol. Plant.* 171 125–136. 10.1111/ppl.13220 32981119

[B73] Xie Z. Wang J. Wang W. Wang Y. Xu J. Li Z. (2021). Integrated analysis of the Transcriptome and Metabolome revealed the molecular mechanisms underlying the enhanced salt tolerance of rice due to the application of exogenous melatonin. *Front. Plant Sci.* 11:618680. 10.3389/fpls.2020.618680 33519878 PMC7840565

[B74] Xiong Q. Q. Shen T. H. Zhong L. Zhu C. L. Peng X. S. He X. P. (2019). Comprehensive metabolomic, proteomic and physiological analyses of grain yield reduction in rice under abrupt drought-flood alternation stress. *Physiol. Plant* 167 564–584. 10.1111/ppl.12901 30561011

[B75] Xu Y. Zou J. Zheng H. Xu M. Zong X. Wang L. (2019). Rna-seq transcriptome analysis of rice primary roots reveals the role of flavonoids in regulating the rice primary root growth. *Genes* 10:213. 10.3390/genes10030213 30871177 PMC6470995

[B76] Yang L. Fountain J. C. Ji P. Ni X. Chen S. Lee R. D. (2018). Deciphering drought-induced metabolic responses and regulation in developing maize kernels. *Plant Biotechnol. J.* 16 1616–1628. 10.1111/pbi.12899 29431900 PMC6097124

[B77] Zhang J. W. Hong-Xia W. U. Dong S. T. (2009). Effects of shading on yield and quality of summer maize. *J. Maize Sci*. 76 819–825. 10.3168/jds.S0022-0302(93)77406-8

[B78] Zhang R. Li S. He J. Liang Y. (2019). Big regulates sugar response and c/n balance in arabidopsis. *Plant Signal. Behav.* 14:1669418. 10.1080/15592324.2019.1669418 31580197 PMC6804704

[B79] Zheng Z. (2009). Carbon and nitrogen nutrient balance signaling in plants. *Plant Signal. Behav.* 4 584–591. 10.4161/psb.4.7.8540 19820356 PMC2710548

[B80] Zinselmeier C. Jeong B. Boyer J. S. (1999). Starch and the control of kernel number in maize at low water potentials1. *Plant Physiol.* 121 25–36. 10.1104/pp.121.1.25 10482657 PMC59374

