# Supplementary material for: Ovary Abortion Induced by Combined Waterlogging and Shading Stress at the Flowering Stage Involves Amino Acids and Flavonoid Metabolism in Maize
Source: Front Plant Sci. 2021 Nov 23;12:778717. doi: 10.3389/fpls.2021.778717 (PMC8649655; doi:10.3389/fpls.2021.778717)
Supplement: Supplementary file 1 [file Data_Sheet_1.zip › Supplementary Figures.PDF]

Supplementary Figures

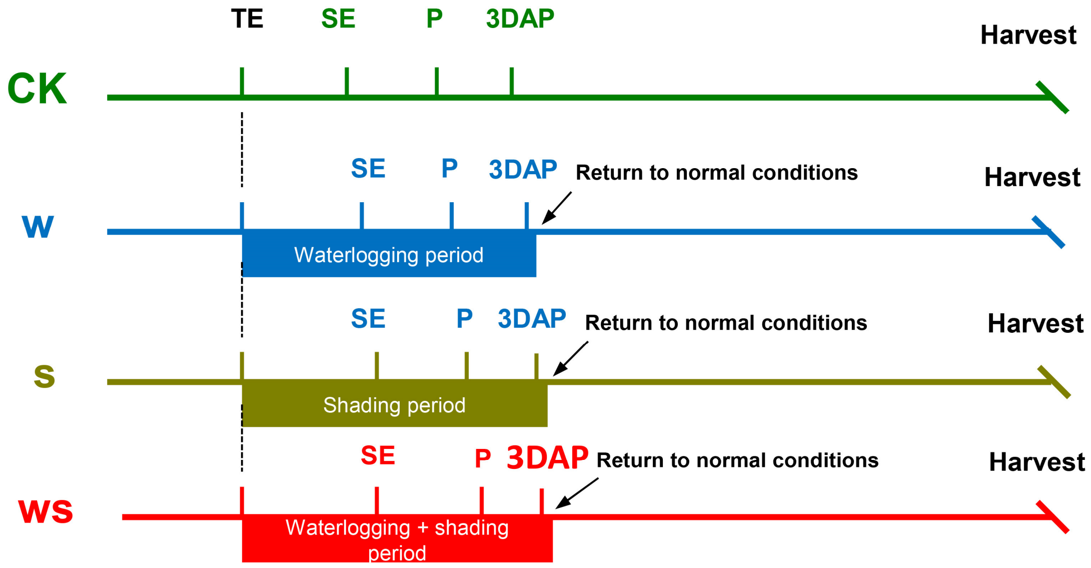

Figure S1. Time course of stress treatment and sampling dates. CK, control; W, waterlogging stress, S; shading stress; WS, combined waterlogging and shading stress; SE, first silk emergence; P, full silk emergence; 3DAP, 3 days after pollination; TE, tassels fully emerged from the whorl (beginning of treatments).

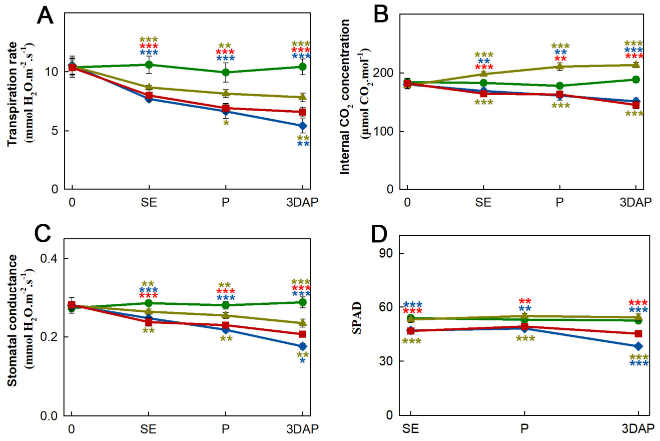

Figure S2. Transpiration rate **(A)**, internal CO_2 concentration **(B)**, stomatal conductance **(C)**, and SPAD value **(D)** of plants in control (CK), waterlogging + shading (WS), waterlogging (W), and shading (S) treatment groups. Colored asterisks above indicate significant differences between control (CK) and treatment groups, colored asterisks below indicate significant differences between WS and W or S groups. Error bars represent ± SD ($n \geq 3$). *P<0.05, **P<0.01, ***P<0.001 (ANOVA and t-test).

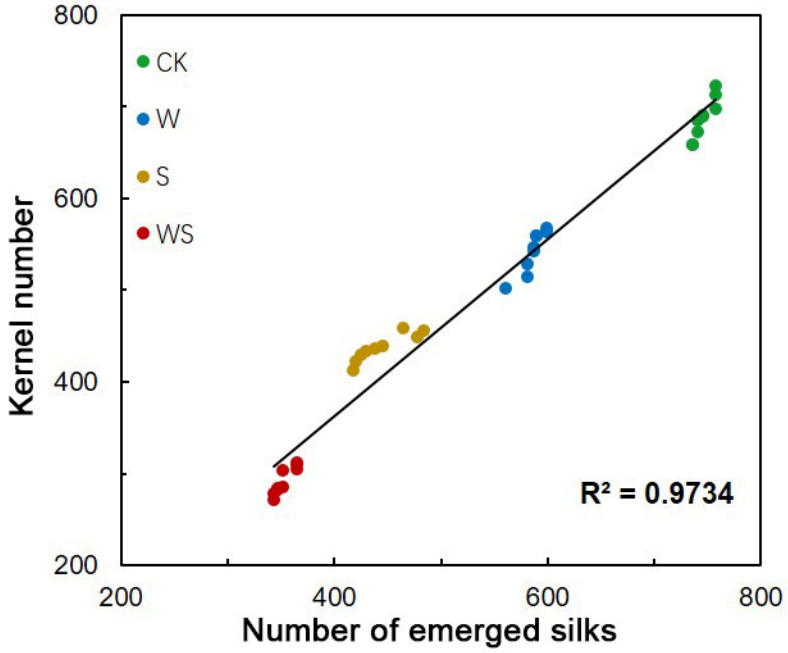

Figure S3. Kernel number as a function of final number of emerged silks per ear. Each circle represents a maize plant. Green, control (CK); blue, waterlogging (W) treatment group; yellow, shading (S) treatment group; red, waterlogging + shading (WS) treatment group.

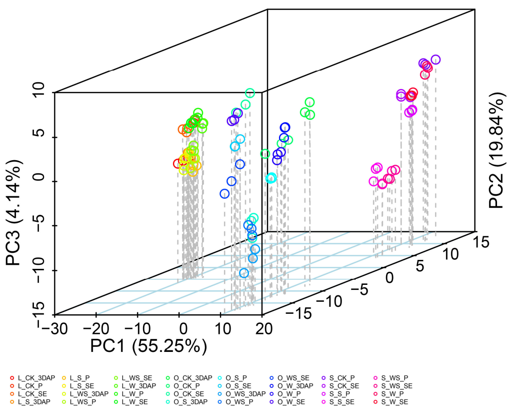

Figure S4. Principal component analyses (PCA) of metabolomics data for leaf, ovary and silks. L (leaf), O (ovary), S (silks).

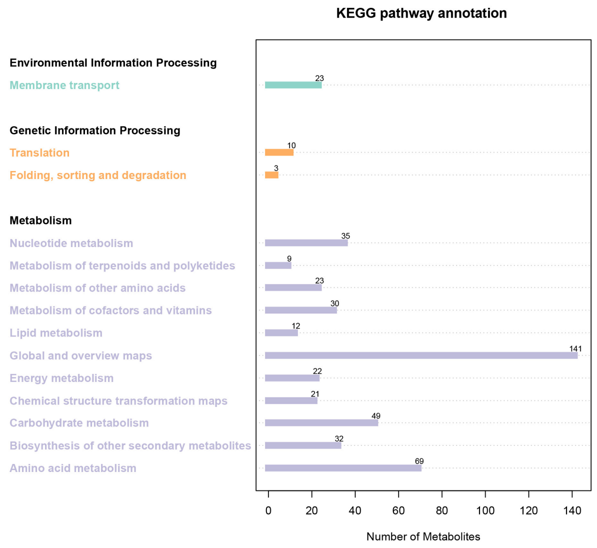

Figure S5. Kyoto Encyclopedia of Genes and Genomes (KEGG) analyses of 430 metabolites.

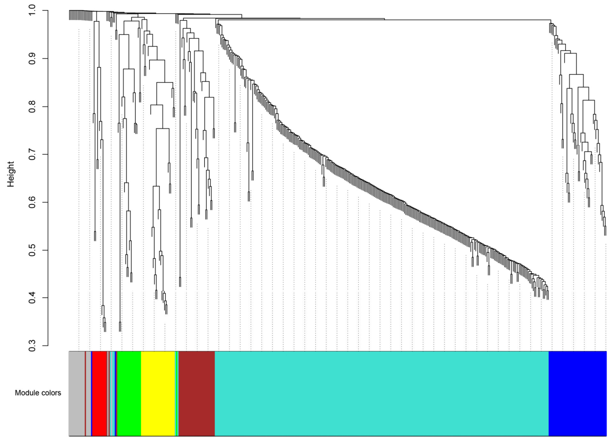

Figure S6. Hierarchical cluster dendrogram based on metabolites identified by WGCNA in all tissues across six co-expression modules.

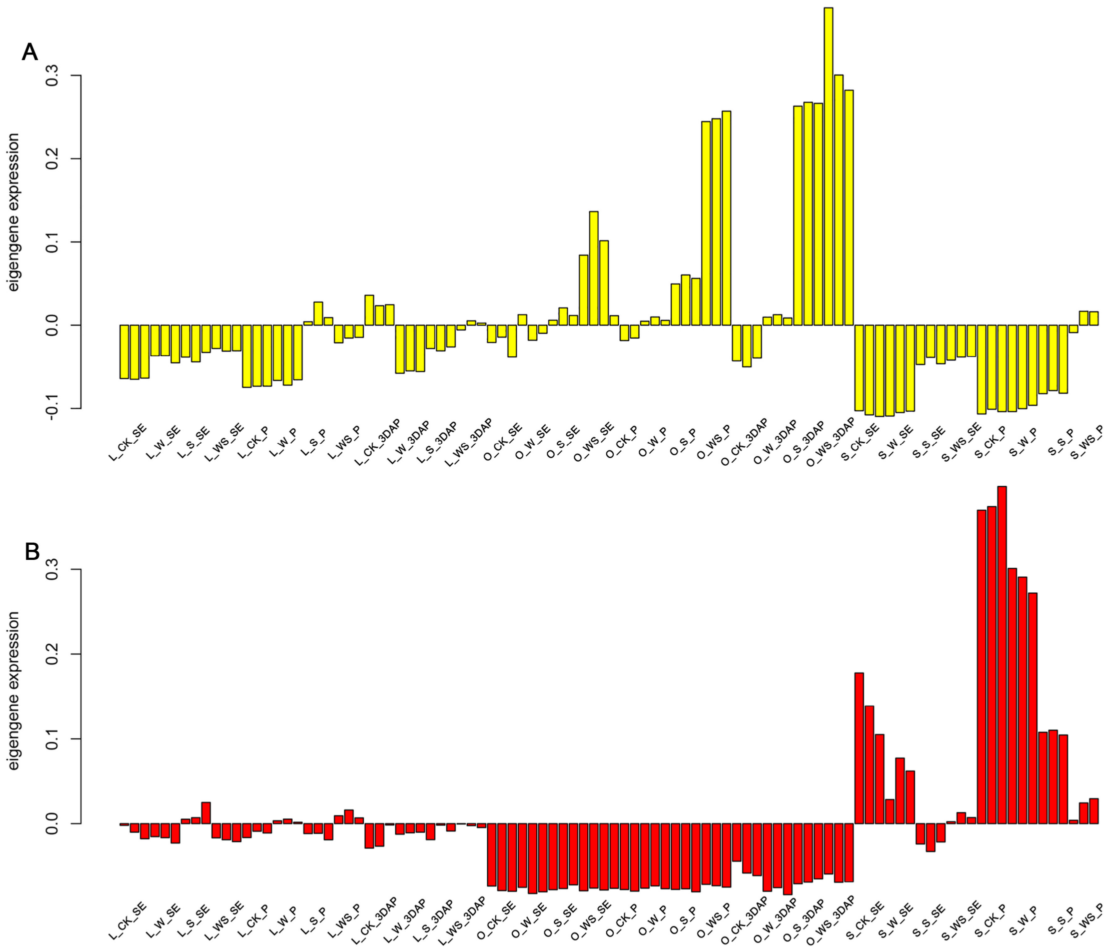

Figure S7. Eigengene expression analyses of metabolites in yellow **(A)** and red **(B)** modules. Each column represents a duplicate.

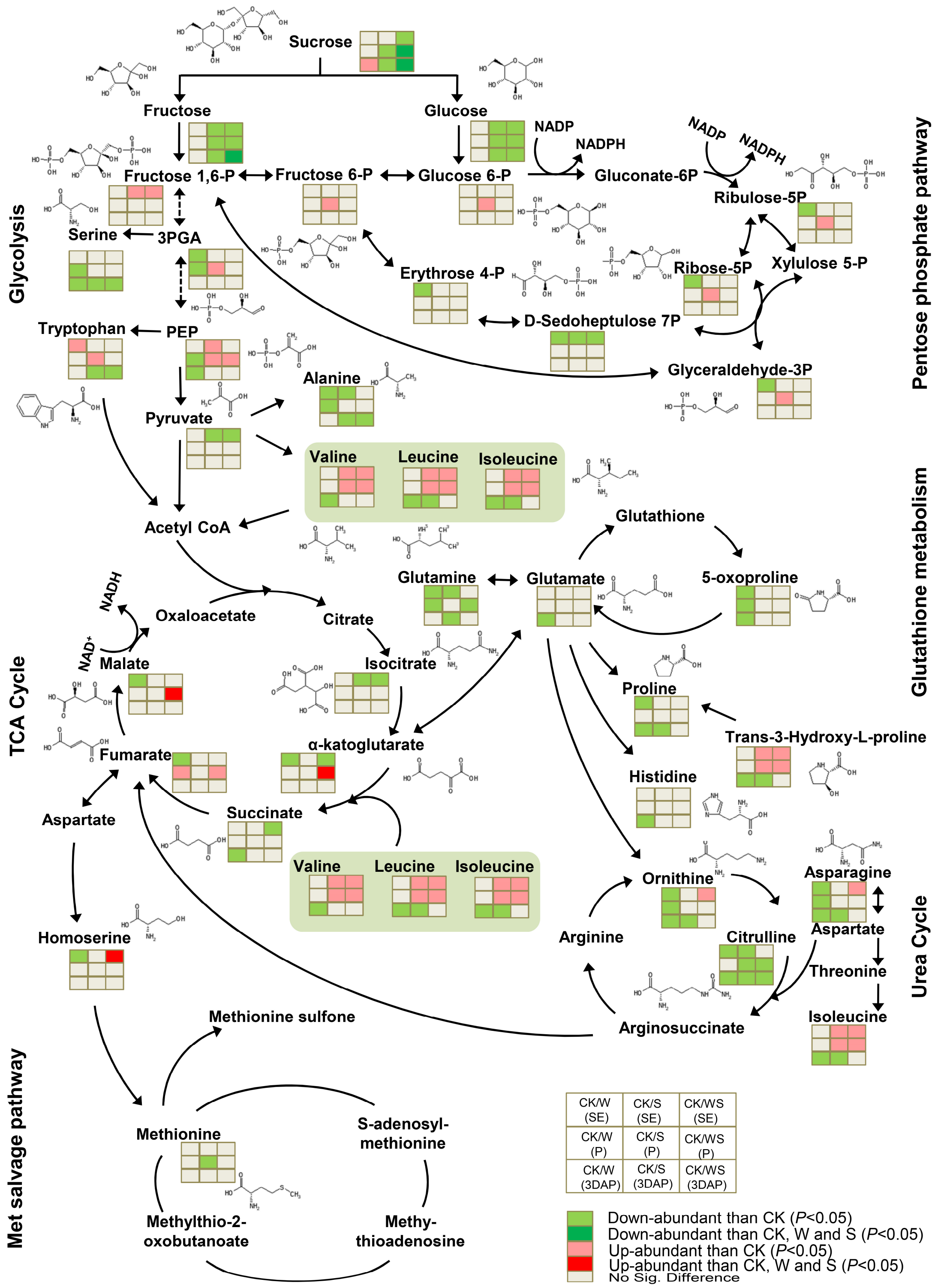

Figure S8. Differences in metabolites involved in carbohydrate and amino acid metabolism in leaves affected by waterlogging + shading (WS), waterlogging (W), and shading (S) treatment at SE (first silk emergence), P (full silk emergence) and 3DAP (3 days after pollination). Grids next to each metabolite represent accumulation of corresponding metabolites in the W, S, and WS groups at each time point. Colors correspond to the significance of the change in accumulation. Light red: more abundant than in the control (*P*<0.05). Deep red: more abundant than in the control, W, and S

($P<0.05$). Light green: less abundant than in the control ($P<0.05$). Deep green: less abundant than in the control, W, and S ($P<0.05$). Light gray: no significant difference compared with control.

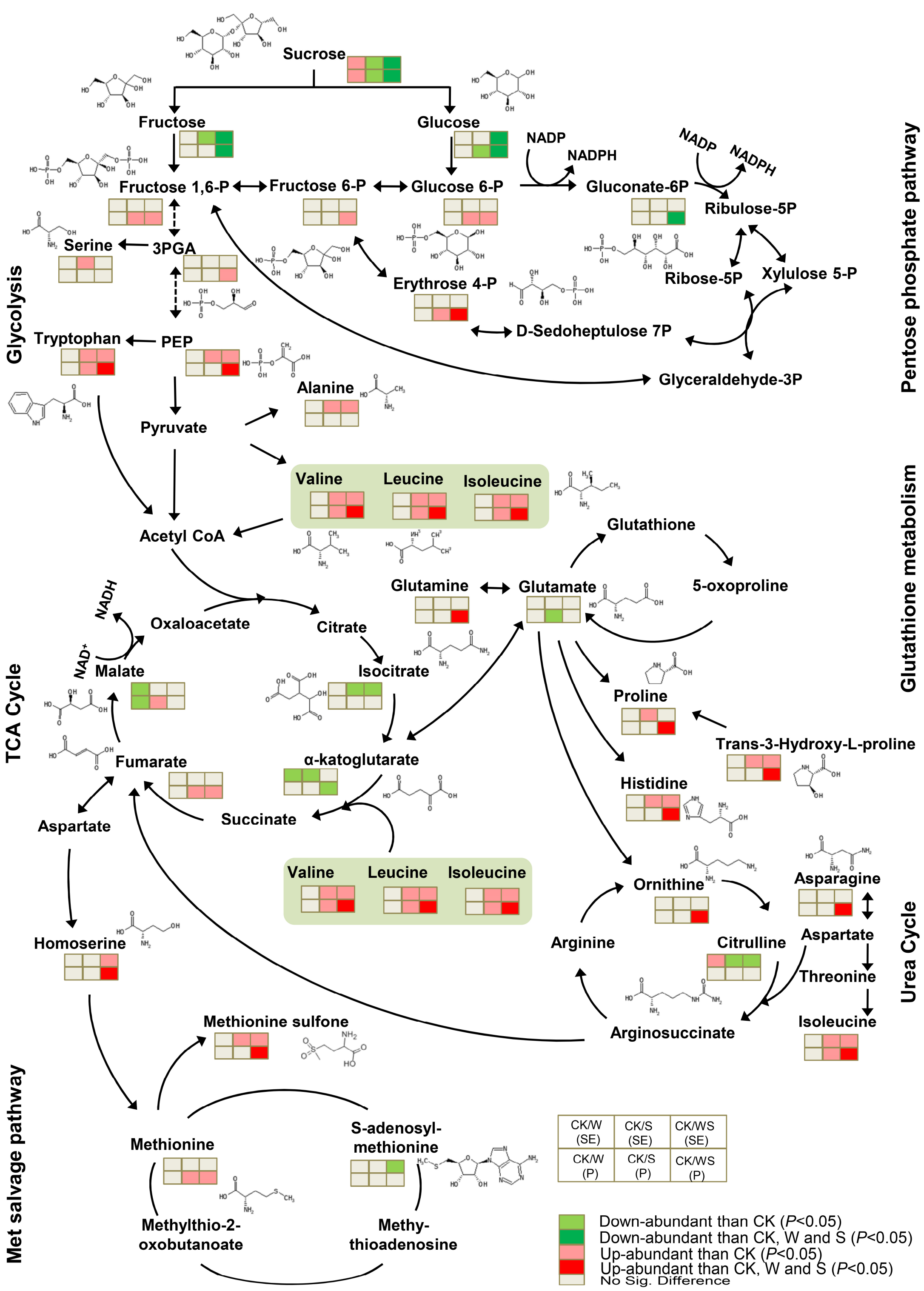

Figure S9. Differences in metabolites involved in carbohydrate and amino acid metabolism in silks affected by waterlogging + shading (WS), waterlogging (W), and shading (S) treatment at SE (first silk emergence) and P (full silk emergence). Grids next to each metabolite represent accumulation of corresponding metabolites in the W, S, and WS groups at each time point. Colors correspond to the significance of the change in accumulation. Light red: more abundant than in the control (*P*<0.05). Deep red: more abundant than in the control, W, and S (*P*<0.05). Light green: less abundant

than in the control ($P<0.05$). Deep green: less abundant than in the control, W, and S ($P<0.05$). Light gray: no significant difference compared with control.
