# Supplementary material for: Ovary Abortion Induced by Combined Waterlogging and Shading Stress at the Flowering Stage Involves Amino Acids and Flavonoid Metabolism in Maize
Source: Front Plant Sci. 2021 Nov 23;12:778717. doi: 10.3389/fpls.2021.778717 (PMC8649655; doi:10.3389/fpls.2021.778717)
Supplement: Supplementary file 1 [file Data_Sheet_1.zip › Supplementary File 1.PDF]

Quasi-Targeted Metabolomics - Materials and Methods

1. Metabolites Extraction

Tissues (100 mg) were individually grounded with liquid nitrogen and the homogenate was resuspended with prechilled 500μL 80% methanol and 0.1% formic acid by well vortexing. The samples were incubated on ice for 5 min and then werecentrifuged at 15000 rpm, 4℃ for 10 min. A some of supernatant was diluted to final concentration containing 53% methanol by LC-MS grade water. The samples were subsequently transferred to a fresh Eppendorf tube and thenwere centrifuged at 15000 g, 4℃ for 20 min. Finally, the supernatant was injected into the LC-MS/MS system analysis.

Each experimental samples were taken equal volume and blended as QC samples.

Blank sample is 53% methanol aqueous solution containing 0.1% formic acid instead of experimental sample， the pretreatment process is the same as the experimental sample.

PS: Liquid sample (100 μL) and prechilled methanol (400 μL) were mixed by well vortexing.

Cell sample (50 μL) and prechilled 80% methanol (200 μL) were mixed by well vortexing, and then sonicated for 6 min. Repeat this step once again, then operate the same steps as above.

2. HPLC-MS/MS Analysis

1）Positive Ion Mode

LC-MS/MS analyses were performed using an ExionLC™ AD system (SCIEX) coupled with a QTRAP® 6500+ mass spectrometer (SCIEX). Samples were injected onto a BEH C8 Column (100×2.1 mm, 1.9μm) using a 30-min linear gradient at a flow rate of 0.35mL/min forthe positive polarity mode. The eluents were eluent A (0.1% Formic acid-water) and eluent B (0.1%Formic acid-acetonitrile).The solvent gradient was set as follows: 5% B, 1 min; 5-100% B, 24.0 min; 100% B, 28.0 min；100-5% B, 28.1 min；5% B, 30 min. QTRAP® 6500+ mass spectrometer was operated in positive polarity mode with Curtain Gas of 35psi, Collision Gas of Medium, IonSpray Voltage of 5500V, Temperature of 500℃, Ion Source Gas of 1：55, Ion Source Gas of2：55.

2）Negative Ion Mode

Samples were injected onto aHSS T3 Column(100 mm×2.1 mm) using a 25-min linear gradient at a flow rate of 0.35mL/minfor the negative polarity mode. The eluents were eluent A (0.1% Formic

acid-water) and eluent B (0.1%Formic acid-acetonitrile). The solvent gradient was set as follows: 2% B, 1 min; 2-100% B, 18.0 min; 100% B, 22.0 min；100-5% B, 22.1 min；5% B, 25 min. QTRAP® 6500+ mass spectrometer was operated in positive polarity mode with Curtain Gas of 35psi, Collision Gas of Medium, IonSpray Voltage of -4500V, Temperature of 500℃, Ion Source Gas of1：55, Ion Source Gas of2：55.

3. Metabolites Identification and Quantification

The detection of the experimental samples using MRM (Multiple Reaction Monitoring) were based on novogene in-house database. The Q3 were used to the metabolite quantification. The Q1, Q3, RT (retention time), DP (declustering potential) and CE (collision energy) were used to the metabolite identification. The data files generated by HPLC-MS/MS were processed using the SCIEX OS Version 1.4 to integrate and correct the peak. The main parameters were set as follows: minimum peak height, 500; signal/noise ratio, 5; gaussian smooth width, 1. The area of each peak represents the relative content of the corresponding substance.

4. Data Analysis

These metabolites were annotated using the KEGG database (http://www.genome.jp/kegg/)、HMDB database (http://www.hmdb.ca/) and Lipidmaps database (http://www.lipidmaps.org/).Principal components analysis (PCA) and Partial least squares discriminant analysis(PLS‐DA) were performed at metaX(a flexible and comprehensive software for processing metabolomics data).We applied univariate analysis (t-test) to calculate the statistical significance (P-value).The metabolites with VIP > 1 and P-value< 0.05 and fold change≥2 or FC ≤0.5 were considered to be differential metabolites.Volcano plots were used to filter metabolites of interest whichbased on Log2(FC) and -log10(P-value) of metabolites.

For clustering heat maps, the data were normalized using z-scores of the intensity areas of differential metabolitesand were ploted by heatmap package in R language.The correlation between differential metabolites were analyzed by cor () inRlanguage (method = pearson). Statistically significant of correlation between differential metabolites were calculated by cor.mtest () in R language. P-value < 0.05 was considered as statistically significant and correlation plots were ploted by corrplot package in R language.The functions of these metabolites and metabolic pathways were studied using the KEGG database.The metabolic pathway enrichment of

Novogene 诺禾致源

differential metabolites were performed, when ratiowere satisfied by x/n > y/N, metabolic pathway were considered asenrichment, when P-value of metabolic pathway < 0.05, metabolic pathway were considered as statistically significant enrichment.

Novogene
