# Supplementary material for: Ovary Abortion Induced by Combined Waterlogging and Shading Stress at the Flowering Stage Involves Amino Acids and Flavonoid Metabolism in Maize
Source: Front Plant Sci. 2021 Nov 23;12:778717. doi: 10.3389/fpls.2021.778717 (PMC8649655; doi:10.3389/fpls.2021.778717)
Supplement: Supplementary file 1 [file Data_Sheet_1.zip › Supplementary Table 1.DOCX]

| Treatment | Light intensity | Air temperature | Relative humidity | CO_2_ concentration |
| --- | --- | --- | --- | --- |
|  | (μmol m^-2^ s^-1^) | (°C) | (%) | (μmol mol^-1^) |
| CK | 1559.3a | 33.85a | 58.0a | 373.61a |
| W | 1557.8a | 33.69a | 59.5a | 378.64a |
| S | 708.8b | 33.16a | 59.5a | 379.41a |
| WS | 704.5b | 32.99a | 60.5a | 380.84a |

**Table S1** Microclimate in experimental field under different treatments

Microclimate data from all treatment groups were recorded every day during the treatment period at 11:00 AM. Different letters within a column indicate significant differences (*P*<0.05) (*n*=10).
